# Development of a High Resolution Virulence Allelic Profiling (HReVAP) Approach Based on the Accessory Genome of *Escherichia coli* to Characterize Shiga-Toxin Producing *E. coli* (STEC)

**DOI:** 10.3389/fmicb.2016.00202

**Published:** 2016-02-23

**Authors:** Valeria Michelacci, Massimiliano Orsini, Arnold Knijn, Sabine Delannoy, Patrick Fach, Alfredo Caprioli, Stefano Morabito

**Affiliations:** ^1^European Reference Laboratory for Escherichia coli, Dipartimento di Sanità Pubblica Veterinaria e Sicurezza Alimentare, Istituto Superiore di SanitàRome, Italy; ^2^Istituto Zooprofilattico Sperimentale dell'Abruzzo e del Molise G. CaporaleTeramo, Italy; ^3^Servizio Informatico, Documentazione, Biblioteca e Attività Editoriali, Istituto Superiore di SanitàRome, Italy; ^4^Platform IdentyPath, Food Safety Laboratory, ANSES, Université Paris-EstMaisons-Alfort, France

**Keywords:** accessory genome, allelic variants, STEC subtyping, phylogenesis, bioinformatics

## Abstract

Shiga-toxin producing *Escherichia coli* (STEC) strains possess a large accessory genome composed of virulence genes existing in multiple allelic variants, which sometimes segregate with specific STEC subpopulations. We analyzed the allelic variability of 91 virulence genes of STEC by Real Time PCR followed by melting curves analysis in 713 *E. coli* strains including 358 STEC. The 91 genes investigated were located on the locus of enterocyte effacement (LEE), OI-57, and OI-122 pathogenicity islands and displayed a total of 476 alleles in the study population. The combinations of the 91 alleles of each strain were termed allelic signatures and used to perform cluster analyses. We termed such an approach High Resolution Virulence Allelic Profiling (HReVAP) and used it to investigate the phylogeny of STEC of multiple serogroups. The dendrograms obtained identified groups of STEC segregating approximately with the serogroups and allowed the identification of subpopulations within the single groups. The study of the allelic signatures provided further evidence of the coevolution of the LEE and OI-122, reflecting the occurrence of their acquisition through a single event. The HReVAP analysis represents a sensitive tool for studying the evolution of LEE-positive STEC.

## Introduction

Human infections with Shiga-toxin producing *Escherichia coli* (STEC) cause a wide range of symptoms including uncomplicated diarrhea, hemorrhagic colitis, and the life-threatening hemolytic uremic syndrome (HUS) (Caprioli et al., 2005). The main virulence feature of STEC is the ability to produce Shiga-toxins (Stx), which interfere with the protein synthesis in the target cells, eventually causing their death (O'Brien and Holmes, 1987). The capacity to produce Stx is acquired through infection with bacteriophages conveying the *stx* genes, which can remain stably integrated into the bacterial chromosome (O'Brien et al., 1984).

In spite of the striking biological effect exerted by the Stx, their sole production seems not to be sufficient for causing the disease, at least the most severe forms. As a matter of fact, only a few STEC serogroups are usually isolated from human cases of severe disease (Nataro and Kaper, 1998; Karmali et al., 2003), which share the presence in the genome of mobile genetic elements (MGEs) encoding robust machineries for the colonization of the host gut (McDaniel and Kaper, 1997; Paton et al., 2001; Morabito et al., 2003; Imamovic et al., 2010; Michelacci et al., 2013). Three Pathogenicity Islands (PAIs) have been described in the genome of such STEC serogroups: the *locus of enterocyte effacement* (LEE) (McDaniel and Kaper, 1997), the OI-122 (Karmali et al., 2003; Morabito et al., 2003), and the OI-57 (Imamovic et al., 2010).

The LEE locus governs the ability to induce the typical “attachment and effacement” (A/E) lesion on the enterocyte. It encodes a type three secretion system, effectors subverting the cell functions related with the cytoskeleton assembly and maintenance, and factors mediating the intimate adhesion of the bacterium to the enterocyte, including the adhesin intimin (McDaniel and Kaper, 1997). The other two PAIs carry genes whose products are also involved in the mechanism of colonization, such as Efa1/LifA, encoded by a gene present in the OI-122 (Morabito et al., 2003), and AdfO (Ho et al., 2008), whose genetic determinant is conveyed by the OI-57 (Imamovic et al., 2010).

During the last decades different authors deployed schemes for the classification of the different STEC types (Griffin and Tauxe, 1991; Nataro and Kaper, 1998; Karmali et al., 2003). One of these schemes groups the STEC strains based on the serogroup, relative incidence of human infections, ability to cause severe diseases, association with outbreaks and presence of virulence-associated MGEs in the genome (Karmali et al., 2003). According to this classification, STEC are divided into seropathotypes (SPTs), identified with letters from A to E in a decreasing rank of pathogenicity. SPT A comprises STEC O157, while SPT B includes the STEC belonging to serogroups different from O157 but causing both sporadic cases and outbreaks of HUS, namely O26, O103, O111, O145, and O121. SPTs A and B share the presence of the LEE, OI-57, and OI-122 PAIs in their genome. The SPT C includes a number of STEC serogroups, including O113 and O91, which apparently do not harbor the LEE locus but are sporadically isolated from severe infections. Finally, STEC included in the SPTs D and E have rarely or never been associated with human disease respectively (Karmali et al., 2003). For the last three SPTs the information on the presence and integrity of the three PAIs are scanty.

The complexity of the STEC virulome is an important source of strain genomic variability, which is further augmented by the existence of multiple allelic variants of the virulence genes. Some of the subtypes of *stx2* have been significantly associated with the most severe infection (Friedrich et al., 2002), while some other subtypes of both *stx1* and *stx2* seemed to be primarily associated with a milder course of the disease or confined to animal hosts (Friedrich et al., 2002; Bielaszewska et al., 2006; Persson et al., 2007; Scheutz et al., 2012). A considerable heterogeneity has also been identified in the DNA sequence of the intimin-coding gene *eae*, leading to the identification of at least 18 intimin types unevenly distributed in the different STEC serogroups (Oswald et al., 2000; Tarr et al., 2002; Ito et al., 2007; Madic et al., 2010).

In the present study we developed an approach to simultaneously identify the presence and the allelic types of a large panel of genes carried by the LEE locus, OI-122, and OI-57 PAIs and used it to study the phylogeny of STEC belonging to SPT A, B and C.

## Materials and methods

### Bacterial strains

A total of 713 *E. coli* strains positive for at least one of the three pathogenicity islands LEE, OI-122, and OI-57 were selected among the isolates present in the culture collections of the Istituto Superiore di Sanità (ISS, Rome, Italy) and the Agence Nationale de Sécurité Sanitaire de l'Alimentation, de l'Environnement et du travail (ANSES, Maisons Alfort, France) and used to identify the alleles of the genes harbored by the three PAIs. The panel comprised 358 STEC strains belonging to serogroups O157 (*n* = 81), O26 (*n* = 32), O111 (*n* = 36), O103 (*n* = 8), O145 (*n* = 8), O121 (*n* = 3), and others (*n* = 190) isolated from unrelated human cases of human infections and from food in Italy and France in the period 2008–2011. Additional 355 *stx*-negative *E. coli* of multiple serogroups and isolated from human, food and animal sources in the same countries and period were included in the study. The O157 STEC strain EDL933 was used as positive control (Supplementary Table 1, Sheet 1).

A population of 318 unrelated STEC strains, part of the panel of strains described above, was used to assess the performance of the HReVAP approach. These included 161 isolates of SPTs A and B and belonging to serogroups O157 (*n* = 81), O26 (*n* = 32), O111 (*n* = 33), O103 (*n* = 5), O145 (*n* = 7) and O121 (*n* = 3) and 157 *eae*-negative strains, of serogroups O91 (*n* = 14), O174 (*n* = 10), O113 (*n* = 9), O104 (*n* = 6), O101 (*n* = 3), O153 (*n* = 3), O21 (*n* = 3), and others (*n* = 109). The study population also included a panel of 36 *stx*-negative *eae*-positive *E. coli*, altogether referred to as EPEC, including the following serogroups: O26 (*n* = 12), O127 (*n* = 5), O55 (*n* = 4), O128 (*n* = 3), O125 (*n* = 3), O111 (*n* = 2), O86 (*n* = 2), and others (*n* = 5).

Finally, 39 out of the 161 SPTs A and B STEC were also subjected to whole genome sequencing, followed by Multi Locus Sequence Typing (MLST) and Whole genome SNP analysis with the aim of comparing HReVAP results with those obtained using these DNA-sequence-based methods. The latter isolates included strains belonging to serogroups O157 (*n* = 16), O26 (*n* = 12), O111 (*n* = 7), O103 (*n* = 2), O145 (*n* = 1), and O121 (*n* = 1).

### Real-time PCR and melting curves analysis

Ninety-one primer pairs were deployed to amplify 100–300 bp fragments from as many genes harbored on the three PAIs LEE, OI-122, and OI-57 (38, 12, and 41 genes from each island, respectively). The genomic sequence of the O157 STEC strain EDL933 (Acc. no. AE005174) was used to design the primer pairs using the Primer-BLAST web-tool available on the NCBI webserver. Some of the primers were degenerated to amplify the target genes in all the STEC strains for which a sequence was available in GenBank. The sequences of primers used in this study and their annealing position on the genomic sequence of EDL933 strain are reported in Table 1.

**Table 1:** **List of primers used in this study**.

**Target pathogenicity island**	**Gene name**	**ORF identifier**	**Primer pair**	**Localization of the PCR product in the genome of EDL933 strain (Acc. no. AE005174)**	**Product length (bp)**
LEE	rorf1	Z5143	TGCCACCCAGAATAGCCCTGC	4691915–4692180	266
			CATCTGCTTTCCATGCCAGCCC		
	espG	Z5142	AGCTGAAGTTGTGGARTTTTTATGC	4691215–4691313	99
			TGTGTCAACRTTAAGGCTGGCA		
	ler	Z5140	CGCCCGACCAGGTCTGCCC	4688879–4689069	191
			GCAGGAAGCAAAGCGACTGCG		
	orf2	Z5139	AATGAGCAGTTCCTTTGCTTC	4688596–4688748	153
			ACGATAACTGARCTGGAAGAYG		
	cesAB	Z5138	GCTCTTCACCATATGTGTACCCC	4688331–4688396	66
			AAACAGATCCTCAGGCGGCAG		
	orf4	Z5137	CTGGTTTACATTGCCCTCCCT	4687810–4687960	151
			AGATTGCTTGCTCTAAGTAGTGC		
	orf5	Z5136	CATTAAGACCAACTCGATAGCCTGG	4687312–4687497	186
			GAACATTTACTGAGGGCGAGGAGG		
	escR	Z5135	TTGCCAGCCTCCAACAAGAATG	4686348–4686439	92
			TCTTATTGGCCTTGGGTATGATG		
	escS	Z5134	CAGTGATAAGTCAGGGCAAGCG	4686113–4686297	185
			GTTCAATTATGTGTGCAAACGTTCTGG		
	escT	Z5133	ACCCAAACCCCATATCACATAAG	4685443–4685591	149
			CGAGATATTACCATTACAAGCC		
	escU	Z5132	ACACTAATCTTCTGAGCCGATGG	4684910–4685199	290
			GAGGTAATGGCTGCAGTGCAGTC		
	rorf3	Z5131	TCTGTTGCTGGTTATATCTTAGCC	4684061–4684174	114
			TCTGAGTTCTACTGCATTTGGTG		
	grlR	Z5129	CACAGAAGCAATATCGCCGCC	4683473–4683549	77
			GCAATGAAGACTCCTGTGGGG		
	grlA	Z5128	ATCTCAAGGCGCTTATAATGC	4682901–4683019	119
			GGTATAAACCTGAGGAGAGCA		
	cesD	Z5127	CCAGGATGGTGGCACATTAGCG	4681955–4682143	189
			CGCTGATGACATAAGCCCAGAGA		
	escC	Z5126	ACCGGAAAACGTCGGGAAC	4681565–4681701	137
			GTCGCTTCTGTTGTCATTTCTG		
	sepD	Z5125	GGATGTCAACGGTTCACCAACGC	4679969–4680129	161
			CGGCCAGGATATTGCAGAGTCTG		
	escJ	Z5124	AGTGGCATCGCCATCACAAG	4679610–4679711	102
			ACGCTGCAATCGATAACACC		
	rorf8	Z5123	CAGCCCCCTCTGCGGCAGG	4679037–4679206	170
			TGGCGAGAGCCCACCTGCAG		
	sepZ	Z5122	TACGGCTCTGGCAGCTCTAGG	4678718–4678808	91
			GCCAGAAGTAATACCCAGGGC		
	orf12	Z5121	GTCATCCTGCGAACGCGCTC	4678032–4678188	157
			CCCTTGCTCCTGAGCGTACG		
	escV	Z5120	GCCAACAACAATATTACCGC	4677681–4677797	117
			TTGATGCGCCTGTCGCTCAG		
	escN	Z5119	CGCCCAAGTAGTGCGTCTCC	4675680–4675765	86
			CATGTTTCCGGCATGTACTGTGG		
	orf15	Z5118	TCGCTGCTTTTGCTCATCAAATG	4674438–4674548	111
			GAGTCGATTGCCTCTAAGCAG		
	orf16	Z5117	AAACAATTCCTCGTAATCTTCCAC	4674052–4674132	81
			CCTCGGGCCGAAGACGACGC		
	sepQ	Z5116	AGTCTGCATTACCCATAAGATC	4673626–4673725	100
			ACAGTTCATTCAGTGGTGCCA		
	cesF	Z5114	GCTCTTGAAGAGCAAACTGACGTG	4671935–4671998	64
			CCAGTATCCATTCATGGCCATCG		
	tir	Z5112	GATCCCGGCGCTGGTGGG	4668929–4668997	69
			CATGGGAGGATTAACGGGGG		
	cesT	Z5111	GGGGTAGCATCATCGAGAGGG	4668459–4668542	84
			GAATGGTGGGCCATATCTGTGC		
	eae	Z5110	CTCATGCGGAAATAGCCGTTA	4667314–4667415	102
			CATTGATCAGGATTTTTCTGGTGATA		
	escD	Z5109	AATGGTGCGATGCGTAATCGGG	4663987–4664092	106
			GATTGAGGCCTTGTTCAAGGGG		
	sepL	Z5108	CATAAACCAGTCAAGTAAAGAGGC	4663194–4663283	90
			CAGGAAGGTATCCAGAAGATCAA		
	espA	Z5107	ATCAGTGCTACTCTGAACATCAGC	4662413–4662628	216
			GGTAATATGTCGAAGGATGAGGTGG		
	espD	Z5106	TGCAGYGCCATTGCTGTTGC	4661487–4661604	118
			GTAAAATTSTTGGYCAGGTCTTTG		
	espB	Z5105	GWYGTYATATCACGCAGACG	4660090–4660258	169
			CGAARACRTTGCCAACAACRRTATCTG		
	cesD2	Z5104	AGCCCTGTTTGGTTACGTGC	4659823–4659923	101
			ATCGGATGGCGGATTGAGAC		
	escF	Z5103	CAGGGTCATTAACCAAATCGGTGC	4659474–4659557	84
			CTCAACAAATGGGTGAAGTAGG		
	orf29	Z5102	GCCTTTACCGCATCATTTTTTC	4659159–4659289	131
			CATAAGCTTCCCAAATATGCRGA		
	espF	Z5101	ACGCACGGCCTGAGGGGC	4658870–4658960	91
			TTCTACACTAGGGCGGCAGC		
OI-122		Z4318	TTTTGCCGGAATACCTCAGG	3924458–3924551	94
			ATGAGAATGCGGTGGAAAAG		
		Z4320	ATGTCGGTTTTTCCGTCCCA	3924862–3924936	75
			TCACGACTGGAGAGCTTTCTG		
	pagC	Z4321	ATCGATATTGCAGATTCACTCC	3925569–3925657	89
			GGCTGATAATCATACGCTATCG		
		Z4322	ATTCCGCACATCTCTGTGGCTA	3926293–3926396	104
			AATGCTGCACTGGCGGTGGT		
		Z4325	TCGACAAAACGCTACAGGAAG	3928384–3928483	100
			CTCAGTGTTCGGTTAAAATGCTC		
	sen	Z4326	TCCTGGATTATTTTCTGCATTTCA	3929758–3929833	76
			ACTATTGCCAAGTACGCCACAA		
		Z4327	CATCTATTTCTCACGCACTGTAG	3930611–3930733	123
			CAATCAGCAGAGTAATGTTGTC		
	nleB	Z4328	CATGTTGAAGGCTGGAASTTTGT	3931502–3931573	72
			CCGCTACAGGGCGATATGTT		
	nleE	Z4329	AGAAGCGTTTGAACCTATTTCCA	3932207–3932289	83
			TTGGGCGTTTTCCGGATAT		
		Z4331	TGGAAACCGTGAGCCCATTC	3933942–3934035	94
			TTCTGCGACCTGACCATCCT		
	efa1	Z4332	TTTTCACCAGTTCATCATACAGG	3934766–3934848	83
			CCATTATAAACATTTGCCAGACC		
	efa2	Z4333	ACTAAGATCAATACAAGGATTCC	3936424–3936496	73
			ATCCATCAGGCCATAGGTG		
OI-57		Z2036	ATCGCCCGTTTGCTGAGCTT	1850106–1850256	151
			ACGTTTTGGCACCAGACCGT		
		Z2037	TTTCCGGTTCCTGTTGCGCT	1851736–1851871	136
			ACCCGCGATTCTGTGAACCA		
		Z2039	TTTGTGATGCGGTGCCTGGT	1853872–1853944	73
			GGGATTCTCTGTATCCGGCGTT		
		Z2045	ATCGGTTCGCCTGGTTGCTT	1855996–1856164	169
			AGGCCGCATTAGGTAAGCTGGT		
		Z2046	AGCTTGCCAATGTCGCAGGA	1856345–1856517	173
			TTCATTGTTCAACCGCCCCG		
		Z2048	TGGCTTTGCCGGAGACAGAA	1857700–1857842	143
			TTTAACCTGCGCCCTGACGT		
	adfO	Z2053	AACTGTCGCCGCAATCCGAA	1860616–1860778	163
			GTCTGGCGCTATTTCCACGACA		
		Z2054	AAGGTCAAGGAGAAGCAGGCT	1861231–1861334	104
			TCTTTCCTCGTTACCAGTGCCGT		
		Z2056	TGACTGGCTGTTGCGTCATGT	1862484–1862594	111
			TGCCAGCACAACACCATTGC		
		Z2057	TATCAAAAGCCGGGGAGCGT	1863371–1863465	95
			TTTTATTGCCAGCCGTCCGGA		
		Z2060	ATGCGGAGCTGCAGAGTGAA	1865516–1865652	137
			TTCTGCCGGTTTTTCGCACG		
		Z2065	CACAGCAACCTGCGCTTGTT	1867369–1867445	77
			TGGCACTGCGCGTTAAACAC		
		Z2066	TTACGGTGCGGCATCGAGAA	1867659–1867735	77
			TGCGCGCCCATGAACTGAAA		
		Z2069	TGTCATACACCACGTCAGCGGT	1868962–1869084	123
			ATGTCAGCAGCCCAAACAGCA		
		Z2071	TTCTGGCGCTGATTGGTGCA	1869583–1869674	92
			ACGGTATGCTGTGGTGTGGT		
		Z2073	ATGCTCTCAGCCATCGCGTT	1871270–1871454	185
			ACATTCGTGCTGGATGCGCT		
		Z2082	AACGCCATCGAGTCGCTGAA	1878129–1878228	100
			TTGCCAGCCACACGACCTTT		
		Z2084	ATGGTTGGGGCAAACTCGCTT	1878830–1878951	122
			AACCAGATGGGCCATGCAGA		
		Z2085	TCTTCGCCGTTGACGTTGGT	1880218–1880319	102
			AAAAGGCGGGGAAGATGGCA		
		Z2086	CCGGTTACTGAGCAGAACACCA	1880974–1881105	132
			TTAACGTCACCTGGAGGCACCA		
		Z2090	ACAGGCGTTGTGGTCCCTTT	1883045–1883229	185
			TGTGTGCAGCTTGTCGGAGA		
		Z2091	TGGGCGTTGCTAATTCAGGCA	1883756–1883832	77
			TCGGGAATGACAACCGTCGGTA		
		Z2093	TGGTGAGCGCGTCACGAAAT	1884483–1884585	103
			TCCGTCACATCAAGCGCACT		
		Z2094	TTCAGGAAATCCGGGCTGCA	1885233–1885302	70
			AGGCCGTTTCCGTTGCTGAT		
		Z2096	ACAAAACGGCGGATGAGCTG	1886138–1886226	89
			ACGCCCTTTGCTGATTGCCA		
		Z2100	ATGAGCGGGTGATTCGTGCA	1889873–1890019	147
			ACAACACCATTGCGCCAGCA		
		Z2101	TTGTTTTGCCTTACCCGCCGAC	1890743–1890847	105
			TAAGTGCCACATCCCGGCGATA		
		Z2102	AACGGCAATTAACGAGGCGCT	1891268–1891390	123
			TCGGTACAATGTGCCAGCCA		
		Z2104	TGCGTTGGCTGCGGCTTTAA	1892470–1892627	158
			TCGCCCTGACTTTGGCACTT		
		Z2108	AAAGGGCAGTACGGCTGTGT	1894801–1894916	116
			AATGCCGAACCACCACGACA		
		Z2109	AAAACCTGGTCCCTGACGCA	1895736–1895839	104
			TTGTTGGTCAGTGCGCCTGA		
		Z2112	ACGTTCACGGGCCATTTCCA	1897682–1897770	89
			TGTTTGCCATGTTCGGCGGT		
		Z2114	TTTCCCGCCGGGCTTTATGT	1899877–1899952	76
			AGACTGCGTTCAAGGGCGTT		
		Z2116	TTCACTGCCGGTTCGCTCAT	1901245–1901344	100
			ATTTTGGATGGAGGGGCCAGCT		
		Z2118	TTTTCAGCTCTCGCATCGGC	1903499–1903600	102
			GCTGGCGAACGCGACAATTA		
		Z2120	AGCGCCTTGTCACGTTCGAT	1904347–1904453	107
			ATCTGCCGTGGTGCCATTCT		
		Z2121	GCTGATGCTTCAACGGCTGAA	1904897–1904991	95
			GCTGACATGCGTAACGAGAATCC		
		Z2131	TTGACTCGCAGCGAAACCGT	1910373–1910508	136
			ATCGCCGCATCCACACGTAA		
		Z2146	AAGTGATGACGGTCGCCACA	1925830–1925936	107
			TGCCGGAGCCTTCATAAGCA		
		Z2150	TGCAGGATGCAGCCAGACTTGA	1928464–1928640	177
			TTTCCAGAGCCACAGCCCTT		
		Z2152	GGCCATCGTTGCTGGTGGATTT	1930016–1930109	94
			TCTGTTCCTGCCCTGCAACA		

Total DNA was extracted from overnight cultures of the strains with the Nucleospin Tissue extraction kit (Macherey-Nagel, Düren, DE).

The Real-Time PCR reactions were performed on the high throughput BioMark Real Time PCR system with 96.96 Genotyping Dynamic Array Chips (Fluidigm, San Francisco, CA), using the EvaGreen DNA binding dye (Biotium Inc., Hayward, CA). The thermal profile was 95°C for 10 min (enzyme activation) followed by 35 cycles of 95°C for 15 s and 60°C for 1 min (amplification step). Finally, a denaturation step was performed and the melting curves of the amplified products were registered. Eight array chips were used to perform the whole panel of reactions, each including a positive template control consisting in the DNA extracted from an overnight culture of the EDL933 strain. In addition to the 91 genes, each sample was also subjected to amplification of the *stx* genes (Perelle et al., 2004) and the *wecA* housekeeping gene, used as marker for *E. coli* species (Forward primer: 5′-CTTTATCTCAGTAGCCTGGG-3′, Reverse primer: 5′-AGGAAGTAACCAAACGGTCC-3′).

### High resolution virulence allelic profiling analysis (HReVAP)

The melting temperatures (Tm) of the amplicons were normalized using the Tm of the PCR products amplified from the positive control strain EDL933 present in each array chip. Normalization values were obtained for each array chip minimizing the mean of the Tm standard deviations for the eight normalized Tm measurements over each gene as well as the overall maximum value of the normalized Tm ranges of all genes.

The Tm frequency distributions for each gene have been calculated by grouping values in 0.05°C intervals. The frequency distributions were then analyzed with the “mix” function of the “mixdist” package in the R software (RTeam, 2014). This function finds Maximum Likelihood estimates for the proportions, means, and standard deviations of a mixture distribution by applying a Newton-type iterative method (RTeam, 2014). The number of Gaussians and the starting parameters were adjusted upon evaluation of several fitting results. In order to limit the degrees of freedom, prior knowledge was applied to the model: in each model fit, the standard deviations (σ) of the Gaussian curves were left variable but constrained to be equal. The following command was used for the analysis: mix[Tm_dat, Tm_par, dist = “norm,” mixconstr(conmu = “NONE,” consigma = “SEQ”)], where Tm_dat is the data frame of the grouped Tm data and Tm_par a data frame of the starting values for the parameters of the distributions. Several normal distributions were observed for each gene at different intervals of temperatures. Before clustering, Tm values were aggregated into numbered classes utilizing the model fitting results to define temperature intervals for distinct alleles. The interval limits were calculated as the points of intersection between two adjoining Gaussian curves with the same standard deviation according to the following equation:

(1) $$\begin{array}{l} T_{int}\left({}^{\circ}\text{​C}\right)=\frac{\mu_{1}^{2}-\mu_{2}^{2}\text{ - 2 }\sigma^{2}\;ln\left(\frac{\pi_{1}}{\pi_{2}}\right)}{\text{2}\mu_{1}\;-\;2\;\mu_{2}}\; \end{array}$$

*T*_*int*_ = Temperature at the point of intersection between Gaussian 1 and 2

μ_*i*_ = Mean temperature of the Gaussian curve *i* = 1, 2

σ = Standard deviation of the Gaussian curves

π_*i*_ = Amplitude of the Gaussian curve *i* = 1, 2

The model fitting used did not prove optimal for allele assignment due to the proximity of many Gaussian curves, with consequent overlap. Therefore, all fits have been revised trying to keep the maximum number of Gaussian curves in the model while aggregating those heavily overlapping. With this procedure, the resolution of the method (capacity of allele distinction) has been lowered in benefit of the precision of the method (convergence of assignments). The Tm intervals identified by peaks in the distributions obtained from the analysis of each gene were used to identify the alleles, which were labeled with numbers in ascending order according to the position of the peak in the temperature distribution (Supplementary Table 2). Each strain was given a numeric allelic signature comprising the alleles of all the genes analyzed.

The *neighbor* software of the Emboss package for samples clustering with default parameters (Rice et al., 2000) was used to compare the allelic signatures and to obtain distance matrices.

Each step of the above described procedure has been implemented in dedicated python classes, including the *neighbor* software that was wrapped together with the TreeGraph software (Stover and Muller, 2010) for graphical trees representation.

The HReVAP software package was deployed and used on the public computational framework ARIES operating on the servers of the Istituto Superiore di Sanità and based on the Galaxy bioinformatics platform (Giardine et al., 2005; Blankenberg et al., 2010; Goecks et al., 2010) (https://w3.iss.it/site/aries/).

The tree files (.tre) produced with the HReVAP clustering algorithm were downloaded and visualized using the FigTree program version 1.4.0 (Drummond et al., 2012).

### Whole genome sequencing of STEC and phylogenetic analyses

Thirty-nine out of the 161 STEC strains used for the HReVAP typing were subjected to whole genome sequencing using the Library Preparation Kit by Kapa Biosystems (Wilmington, MA, USA) and a paired end 100 bp protocol on an Illumina HiSeq2500 instrument in fast run mode according to manufacturers' instructions. The sequencing reads have been uploaded in the EMBL-ENA sequence database (EMBL European Nucleotide Archive Study accession no. PRJEB11886). The raw reads were trimmed to remove the adaptors and to accept 27 as the lowest Phred value and assembled using the *de novo* assembly tool Edena v3 (Hernandez et al., 2008). The contigs were subjected to *in silico* Multi Locus Sequence Typing (MLST) with the protocol described by Wirth and colleagues (Wirth et al., 2006). Whole genome Single Nucleotide Polymorphism (WG-SNP) analysis was performed using the ksnp3 pipeline (Gardner et al., 2015), using 19 as kmer size. The optimum value for the kmer size was selected as that producing the highest number of unique kmers of the median length in all the genomes of the dataset and it was calculated by using the kchooser tool included in the ksnp3 pipeline. All the bioinformatics analyses were performed through the ARIES webserver (https://w3.iss.it/site/aries/).

## Results

### HReVAP: identification of the alleles

The analysis of the 91 genes conveyed by the three pathogenicity islands LEE, OI-122, and OI-57 allowed identifying a total of 476 alleles (Supplementary Table 1, Sheet 1 and Supplementary Table 3). Each gene displayed 2 to 10 different alleles in the study population.

The *eae*-positive strains included in the panel exhibited the presence of 32 out of the 38 LEE-harbored genes on average, while the strains positive for the marker of OI-122, *efa1-lifA*, were positive for the majority of the 12 genes selected on this PAI (10.8 genes on average). The OI-57 showed the widest variability. As a matter of fact, 10.4% of the isolates proved positive for 1 to 10 genes of PAI OI-57, 30.6% were positive for 11–20 genes, 26% fell in the range of 21–30 genes detected and 33% gave positive result for more than 31 out of the 41 targets considered (Supplementary Table 1, Sheet 1).

**Figure 1:**
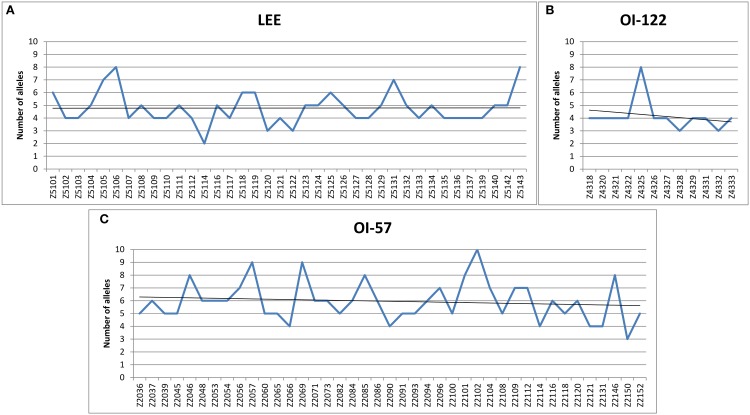
**Allelic variability of the PAIs assayed**. Number of alleles identified for each of the ORFs assayed and trend lines. **(A)** Alleles identified in the ORFs harbored by the LEE locus. **(B)** Alleles identified in the ORFs harbored by the OI-122. **(C)** Alleles identified in the ORFs harbored by the OI-57.

The genes conveyed by the LEE and the OI-122 islands showed a mean number of alleles of 4.79 (range: 2–8 alleles; median = 5) and 4.17 (range: 3–8 alleles; median = 4) for each gene, respectively, while those part of the OI-57 were the most variable, displaying a mean value of 5.95 alleles each (range: 3–10 alleles; median = 6) (Figure 1 and Supplementary Table 3). Interestingly, the LEE locus displayed a uniform allelic variation throughout its whole length while the OI-122 and the OI-57 appeared to have a slightly higher number of alleles in the leftmost part (Figure 1).

### HReVAP performance: amplification of the LEE, OI-122, and OI-57 targets in STEC and EPEC

**Figure 2:**
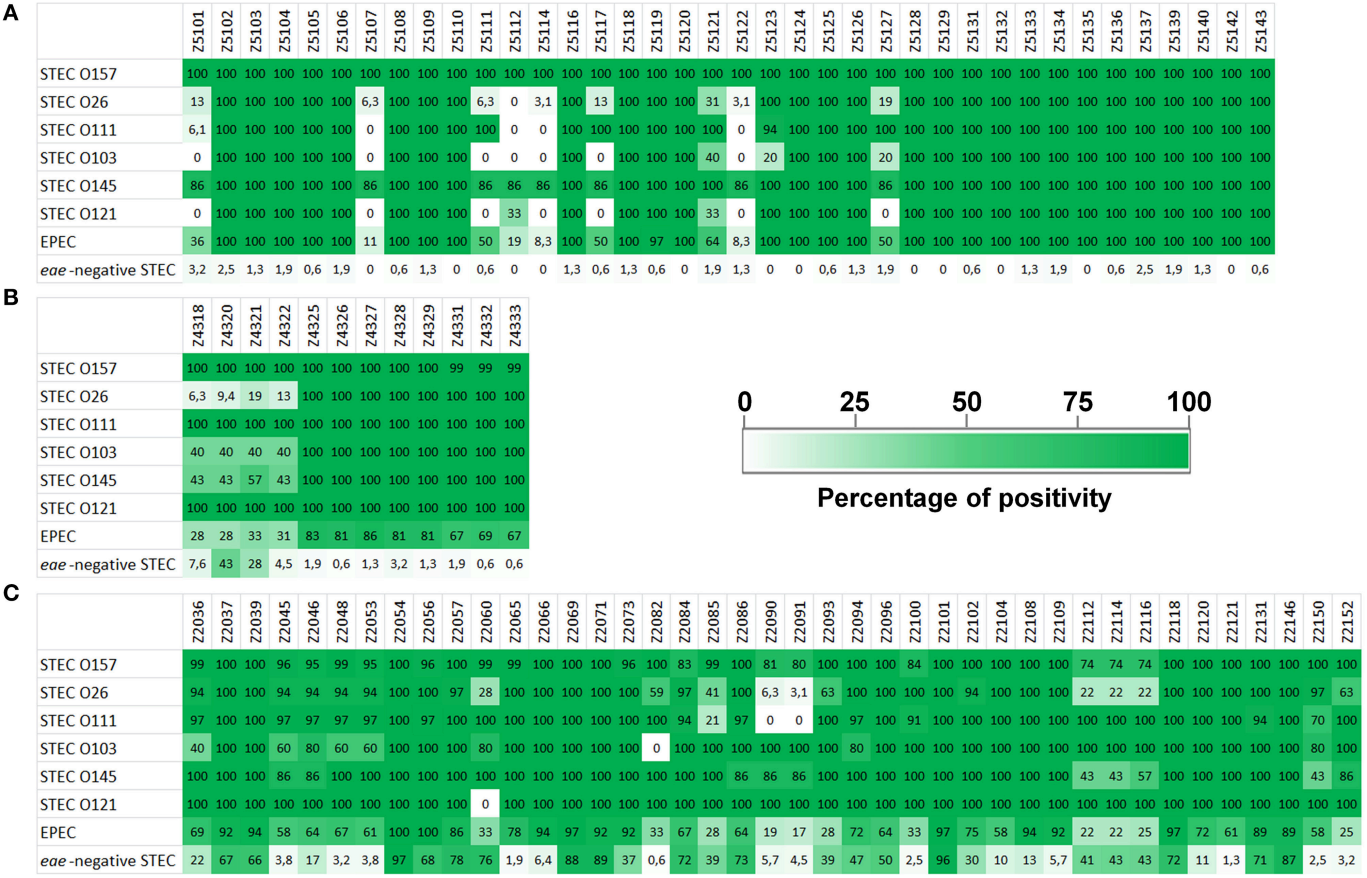
**Amplification of HReVAP targets in STEC and EPEC strains**. The results are reported as percentage of positive strains for different groups of samples, according to the color scale reported in the figure legend. **(A)** Results of the ORFs harbored by the LEE locus. **(B)** Results of the ORFs harbored by the OI-122. **(C)** Results of the ORFs harbored by the OI-57.

All the LEE-genes could be amplified in the vast majority of the STEC strains belonging to SPTs A and B (Figure 2A and Supplementary Table 1, Sheet 2). In detail, no negative results were obtained for all the O157 and O145 strains tested, with the only exception of one O145 strain, negative for eight targets. Nine LEE-borne genes, namely the open reading frames (ORF) Z5101, Z5107, Z5111, Z5112, Z5114, Z5117, Z5121, Z5122, and Z5127 were more variable in the STEC serogroups other than the O157 and O145, with more than 60% of the STEC O26 strains tested producing no amplicons. The same nine targets could not be amplified in all the STEC O103 and O121 strains, with a few exceptions (Figure 2A). Four of these nine gene targets (Z5107, Z5112, Z5114, and Z5122) could not be amplified from the whole panel of the STEC O111, while ORF Z5101 gave positive result in only two strains of this serogroup.

As for the OI-122 PAI, all the 12 ORFs selected were detected in the whole panel of STEC O157, O111, and O121 strains, with the only exception of one O157 strain, which was negative for the three ORFs Z4331, Z4332, and Z4333 (Figure 2B and Supplementary Table 1, Sheet 2).

The STEC strains belonging to serogroups O145, O103, and O26 showed different amplification profiles, with the four ORFs Z4318, Z4320, Z4321, and Z4322 negative in 60% of the O103 strains and in the majority of O145 and O26 strains (Figure 2B).

The HReVAP typing of the STEC and EPEC confirmed the highest degree of variation of PAI OI-57. In particular, ORFs Z2112, Z2114, and Z2116 could not be amplified in many STEC O26, O157, and O145 while ORFs Z2090 and Z2091 were not detected in all the STEC O111, in the majority of STEC O26 strains, and in some STEC O145 and O157. Finally, the ORF Z2085 was frequently negative in STEC O111 and O26 (Figure 2C and Supplementary Table 1, Sheet 2).

Unexpectedly, the *eae*-negative STEC strains tested also showed positivity to many targets of the PAI OI-57 (Figure 2 and Supplementary Table 1, Sheet 3). In particular, two ORFs, Z2054, and Z2101, were positive in more than 95% of the strains tested, while genes Z2037, Z2039, Z2056, Z2057, Z2060, Z2069, Z2071, Z2084, Z2086, Z2096, Z2118, Z2131, and Z2146, were positive in more than 50% of the population assayed.

As a whole, a mean of 15.9 targets out of the 41 selected for OI-57 were present in the panel of *eae*-negative STEC strains (range: 5–39; median = 15) (Supplementary Table 1, Sheet 3).

The EPEC strains assayed provided different amplification patterns (Figure 2 and Supplementary Table 1, Sheet 4). As expected, the LEE-borne ORFs and the OI-122 targets followed a pattern of positivity to PCR similar to that displayed by the STEC belonging to SPTs A and B, although with some variation. The OI-57 was present in all the EPEC strains tested but showed two regions of major variability encompassing the ORFs Z2090-Z2093 (negativity range: 72.2–83.3%) and Z2112-Z2116 (negativity range: 75–77.78%) (Figure 2 and Supplementary Table 1, Sheet 4).

### HReVAP typing: allelic variability of the LEE, the OI-122, and the OI-57

The allelic variability of the genes harbored by the three pathogenicity islands LEE, OI-122, and OI-57 has been investigated in the same study population used to assess the HReVAP performance.

**Figure 3:**
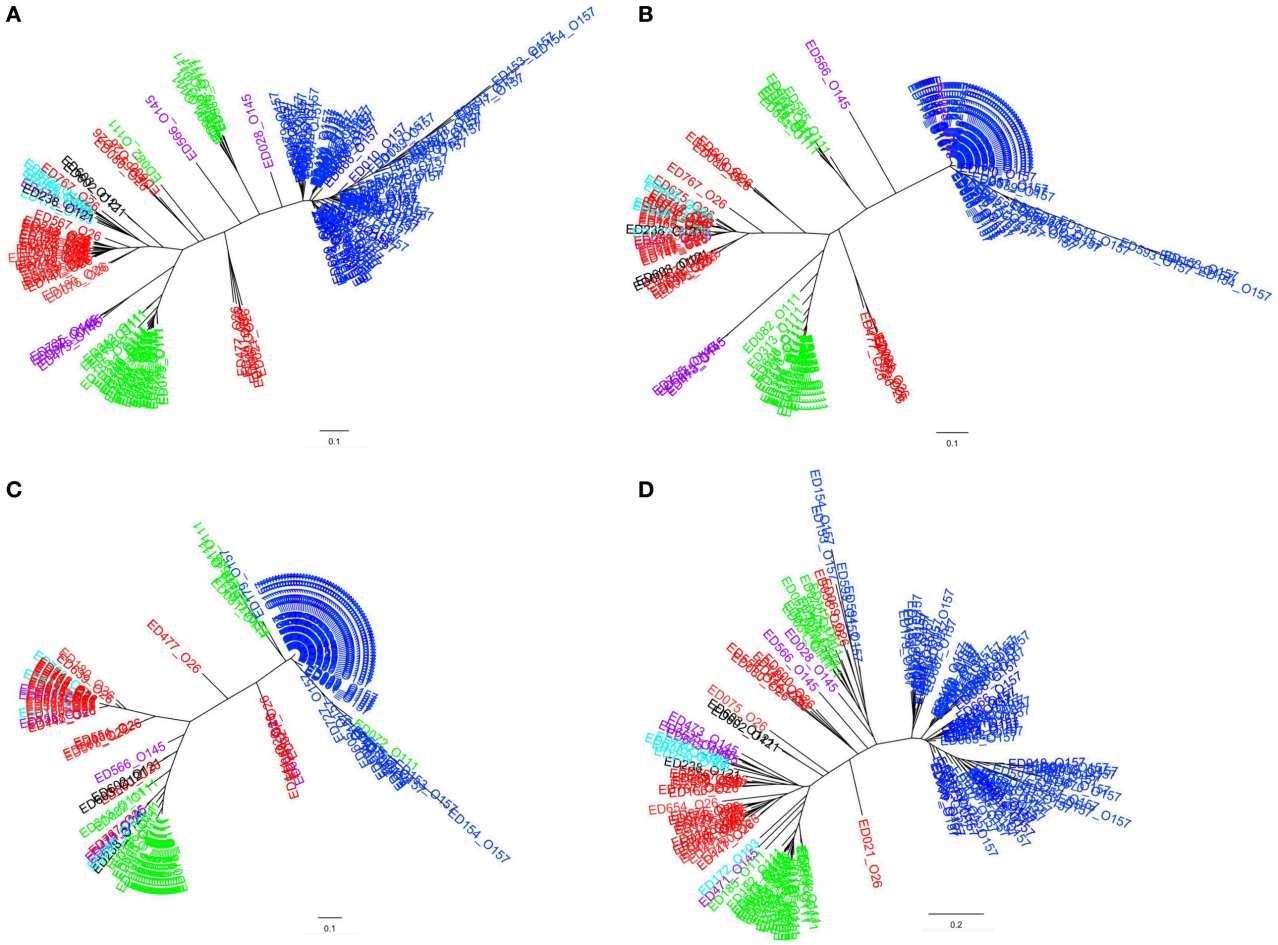
**Clustering of the allelic signatures obtained with the HReVAP from STEC strains belonging to SPTs A and B**. The different serogroups are labeled according to the following color legend: dark blue for O157, red for O26, green for O111, pale blue for O103, purple for O145, and black for O121. **(A)** Dendrogram of the allelic signatures from all the 91 ORFs. **(B)** Dendrogram of the allelic signatures obtained with the ORFs of the LEE locus. **(C)** Dendrogram of the allelic signatures obtained with the ORFs of the OI-122. **(D)** Dendrogram of the allelic signatures obtained with the ORFs of the OI-57.

The clustering of the allelic signatures of the STEC strains of SPTs A and B produced a dendrogram whose branches segregated with the serogroups, with a few exceptions (Figure 3A). In particular, the cluster formed by STEC O157 strains appeared clearly distinct from the others and much more homogeneous, while the strains belonging to O111 and O26 serogroups were divided in two and three distinct clusters, respectively. Similar results were obtained when the cluster analysis was carried out separately using the allelic signatures produced with the ORFs of the LEE locus (Figure 3B) and of the PAI OI-122 only (Figure 3C). Such dendrograms displayed the same topology of that produced when the alleles of the complete ORFs panel were used but showed a lower intra-cluster resolution. More complex results were instead obtained from the HReVAP analysis of the genes conveyed by the OI-57, reflecting the highest variability observed in the ORFs of this PAI (Figure 3D). Even if the main groups corresponding to STEC serogroups O157, O111, and O26 could still be detected, the overall topology showed wider and less defined branches.

**Figure 4:**
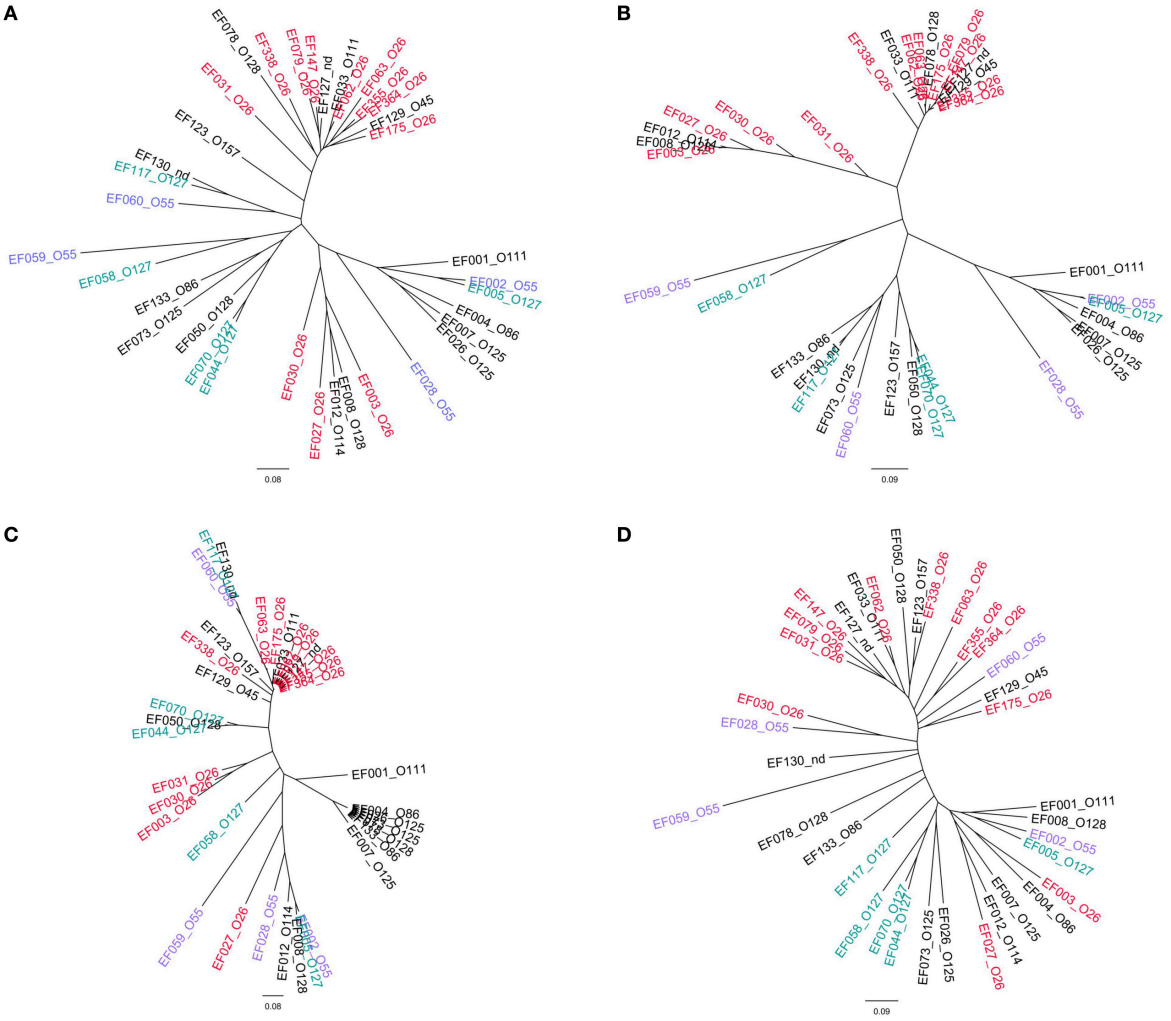
**Clustering of the allelic signatures obtained with the HReVAP from EPEC strains**. The most represented serogroups are labeled according to the following color legend: red for O26, violet for O55, and blue-green for O127. **(A)** Dendrogram of the allelic signatures from all the 91 ORFs. **(B)** Dendrogram of the allelic signatures obtained with the ORFs of the LEE locus. **(C)** Dendrogram of the allelic signatures obtained with the ORFs of the OI-122. **(D)** Dendrogram of the allelic signatures obtained with the ORFs of the OI-57.

The topology of the dendrograms obtained with the EPEC isolates resembled that of those produced with the STEC of SPTs A and B allelic signatures. However, the higher variability of EPEC, together with the lower number of isolates tested, caused the output to be less definite (Figure 4).

**Figure 5:**
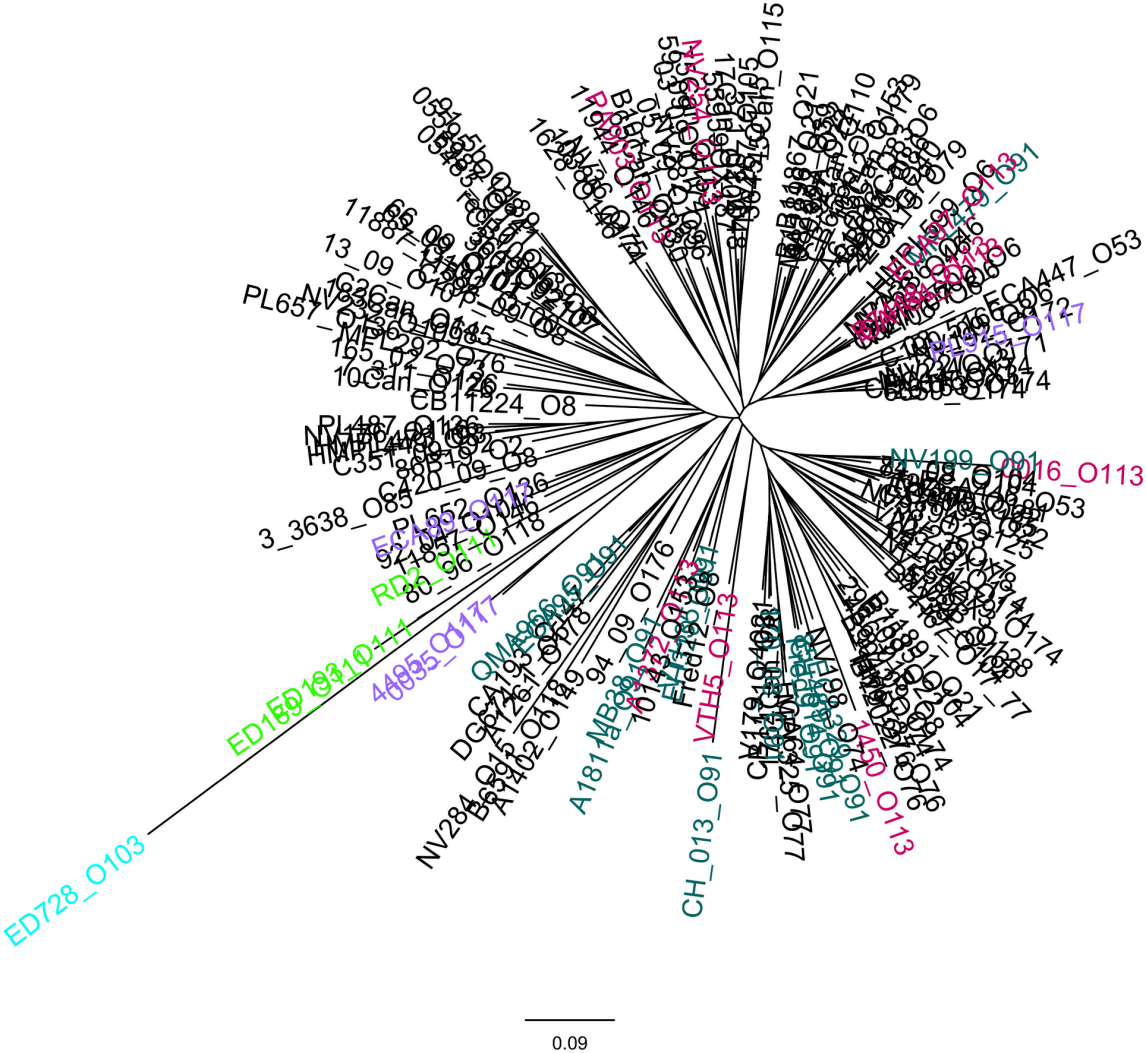
**Clustering of the allelic signatures obtained with the HReVAP from the *eae*-negative STEC strains**. Dendrogram of the allelic signatures obtained with the ORFs of the OI-57. The most represented serogroups are labeled according to the following color legend: crimson for O113, green for O111, pale blue for O103, orange for O91, and brown for O117.

As for the *eae*-negative STEC strains, the cluster analysis of the allelic signatures was carried out exploiting the observed positivity to many of the ORFs of the OI-57. Although based on a smaller number of targets, this analysis showed a massive variability in the allelic signatures, yet able to distinguish and group different populations of strains (Figure 5).

### Comparison between HReVAP and the DNA sequence-based MLST and whole genome-SNP analyses

**Figure 6:**
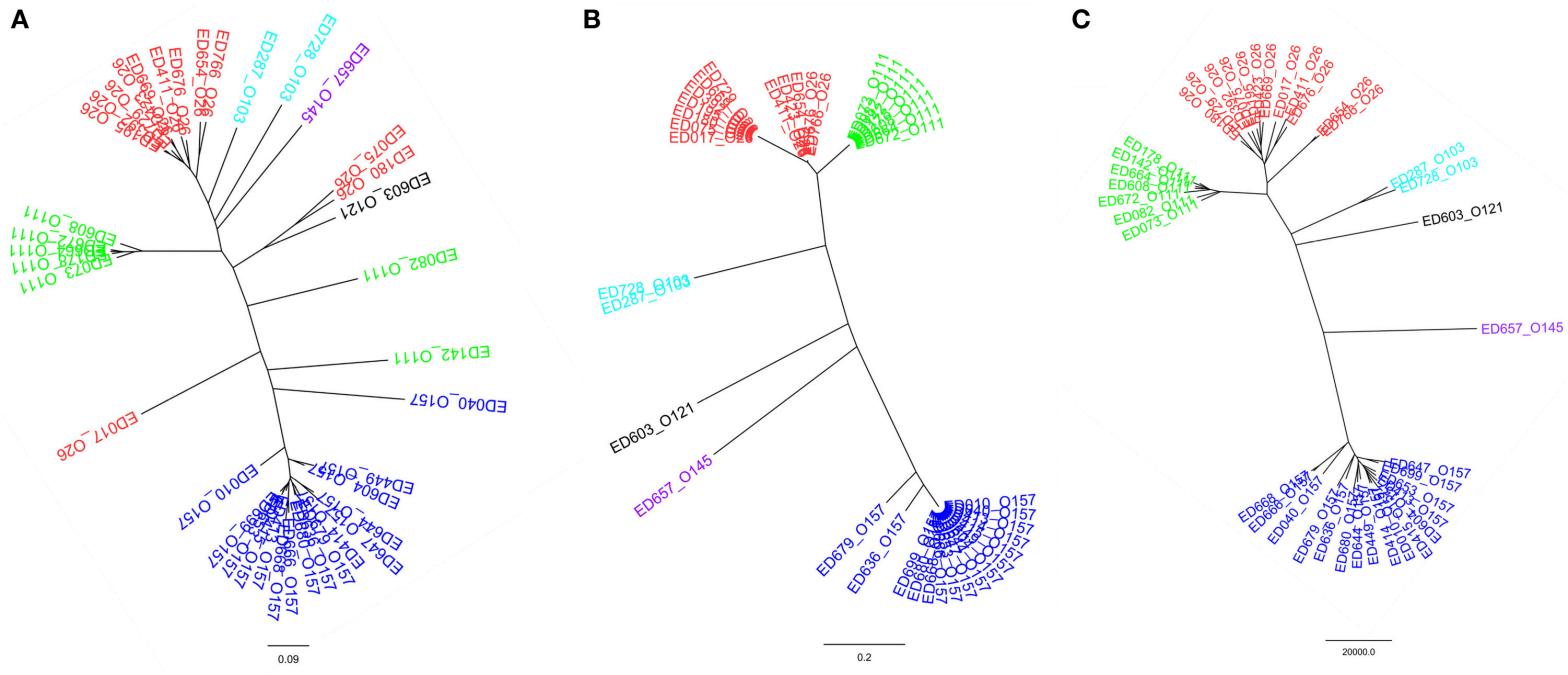
**Comparison of the dendrograms obtained by HReVAP, MLST, and WG-SNP typing**. The different serogroups are labeled according to the following color legend: dark blue for O157, red for O26, green for O111, pale blue for O103, purple for O145, and black for O121. **(A)** Dendrogram of the allelic signatures obtained with the HReVAP typing. **(B)** Dendrogram obtained from the MLST typing. **(C)** Dendrogram obtained from the WG-SNP typing.

Thirty-nine STEC strains were used to compare the results of the HReVAP with those produced by DNA sequence-based typing techniques. The strains were either analyzed with the HReVAP or their genomes subjected to *in silico* MLST and WG-SNP analysis. The comparison showed that the HReVAP (Figure 6A) had a much higher discriminatory power than the MLST (Figure 6B) and produced a dendrogram similar to that produced with the WG-SNP (Figure 6C). Apparently, the topology of the HReVAP dendrogram allowed identifying differences within the serogroups, that were not visible in the WG-SNP-based dendrogram.

## Discussion

The detection of the enzyme isoforms or of the polymorphisms in the genomes of pathogenic microorganisms has been for a long time the basis for the identification of molecular profiles of isolates. Bacterial subtyping has been largely used in research studies on the evolution of bacterial pathogens since the first development of typing methods such as the multi locus enzyme electrophoresis (Selander and Levin, 1980; Selander et al., 1986; Donkor, 2013) and the pulsed field gel electrophoresis (Arbeit et al., 1990), followed by the elaboration of schemes for the identification of the allelic forms of genes as in the multi-locus sequence typing (MLST) (Maiden et al., 1998). Moreover, molecular typing of microorganisms soon demonstrated its great potential in the control of infectious diseases through the implementation of surveillance programs aiming at limiting the burden of infections (Swaminathan et al., 2001). Nowadays, molecular subtyping of bacteria can benefit from cutting edge technologies such as the next generation sequencing (NGS) allowing the detection of whole genome single nucleotide polymorphism (SNP) (WG-SNP) (Kuroda et al., 2010; Vogler et al., 2011; Joensen et al., 2014; Dallman et al., 2015b).

WG-SNP has been successfully used to define a typing scheme for the surveillance of *Listeria monocytogenes* infections (Commission Decision, 2010). However, its application to bacterial pathogens such as *E. coli*, although advisable, is still under debate given the extensive genomic variability of this bacterial species. The development of WG-SNP-based typing concepts for *E. coli* has been attempted by several authors and proved successful for some STEC serogroups such as O157 and, to a lesser extent, O26 (Dallman et al., 2015a,b,c; Holmes et al., 2015; Jenkins et al., 2015). However, a single approach successfully applicable to all the STEC serogroups has not been developed yet.

We have deployed a typing scheme for STEC based on the evaluation of polymorphisms in the sequence of a large panel of virulence genes through the determination of the melting temperature of Real-Time PCR amplicons. Such an approach originated from multiple considerations. The virulence genes, part of the accessory genome, have a higher variability than the rest of the genome. Such an increased variability would reduce the number of targets needed for the phylogenetic analysis. Additionally, comparing the allelic combinations of the fraction of genome shared by all the members of a pathotype (e.g., STEC) should overcome the need of finding an appropriate reference or setting a threshold for the diversity, which would introduce a bias in the evaluation of clusters. These aspects both represent limitations of the currently described whole genome sequence-based methods for *E. coli* (Dallman et al., 2015a,b,c; Holmes et al., 2015; Jenkins et al., 2015). Finally, the use of the widely diffused Real Time PCR to obtain the strains' signatures makes it not necessary to resort to NGS, which is only available in reference laboratories and requires skills and knowledge of the downstream bioinformatics applications that might be unavailable in most of the front-line laboratories.

The rationale behind the proposed concept resides in the many studies published on the allelic variants of known virulence genes of STEC, such as the Shiga-Toxin-coding genes (Friedrich et al., 2002; Bielaszewska et al., 2006; Persson et al., 2007; Scheutz et al., 2012) and the *eae* gene (Oswald et al., 2000; Tarr et al., 2002; Ito et al., 2007; Madic et al., 2010), as well as the more recently described *subAB* and *toxB* genes (Tozzoli et al., 2010; Michelacci et al., 2013, 2014). All the mentioned papers described the association between specific alleles and sub-populations of STEC strains. We investigated the allelic forms of 91 virulence genes conveyed by the three main MGEs associated with STEC pathogenicity, namely the LEE, the OI-122, and the OI-57 (McDaniel and Kaper, 1997; Karmali et al., 2003; Imamovic et al., 2010) and used the obtained allelic signatures to investigate the phylogenesis of STEC.

The whole process, termed High Resolution Virulence Allelic Profiling (HReVAP), allowed us to identify a range of 2–10 allelic forms for each of the 91 ORFs, resulting in the impressive number of 476 total alleles generating a high number of unique allelic signatures.

The HReVAP clustered the LEE-positive STEC strains into groups approximately segregating with the serogroup, providing an indication that the allelic signatures were not randomly assigned to the isolates. Additionally, the analysis identified different subpopulations within the serogroups and also showed variability within each of the populations identified (Figure 3). This finding was not unexpected, since all the strains used in the test panel were epidemiologically unrelated, and at the same time provided an indication that the HReVAP might also be successful in identifying clusters of related strains such as those derived from an outbreak.

The HReVAP produced allelic signatures also with *eae*-negative STEC. However, the dendrogram obtained with these strains had a less resolved topology (Figure 5). An explanation of this result resides either in the lower number of genes these isolates are positive for or in the low number of strains in each serogroup, which in some cases only included one isolate. Nevertheless, the finding that at least part of this PAI was frequently present in *eae*-negative STEC is interesting and constitutes the first report of the presence of this PAI, or its remnants, in this group of STEC.

The HReVAP analysis also proved useful in following the evolution of the single MGEs considered for the typing scheme. As a matter of fact, we could visualize a similar pattern of variation in the allelic signatures obtained considering the ORFs of the LEE locus and the OI-122 (Figures 3B,C). This result indicates that the two MGEs underwent similar evolutionary pathways and supports the previous hypothesis about their common acquisition through a single event of horizontal gene transfer in certain STEC and EPEC strains (Morabito et al., 2003).

Our results showed that the OI-57 had the greatest genetic variability, displaying the highest number of alleles on average for all the ORFs considered (Figure 1C and Supplementary Table 3). Additionally, the analysis of the allelic signatures obtained considering the OI-57 ORFs produced dendrograms with the most dispersed topology (Figures 3D, 4D). These observations suggest that this MGE could have been acquired at an early stage of the evolutionary pathway that led to the emergence of STEC. Additionally, since the LEE-negative STEC investigated were also positive for many of the OI-57-related ORFs considered in this study, it can be hypothesized that such an island could be a common heritage of STEC independently of the presence of the LEE locus.

Finally, the comparison of the performance of the HReVAP with that obtained with other comparative genomic tools such as the MLST and the WG-SNP analysis substantiates the robustness of the HReVAP in identifying LEE-positive STEC populations with a much higher resolution with respect to the MLST and a comparable level of discrimination to that of the WG-SNP.

In conclusion, the HReVAP approach demonstrated good sensitivity and high resolution in the molecular characterization of STEC, particularly for the LEE-positive strains. Moreover, the incredibly large virulome of pathogenic *E. coli* offers the opportunity to refine the HReVAP typing strategy for other STEC groups, such as the LEE-negative isolates, or even to extend it to other *E. coli* pathotypes by integrating the panel of targets.

Further work is in progress to assess the use of HReVAP as an effective tool for the surveillance of STEC infections and to obtain the allelic signatures from whole genome sequences in order to make this technique a cross-generational tool connecting the Real-Time PCR and the NGS-based applications.

## Author contributions

VM conceived the experimental design and drafted the manuscript, MO developed the scripts for HReVAP clustering, and critically revised the manuscript, AK developed and applied the scripts for the extraction of the allelic signatures of the HReVAP and critically revised the manuscript, SD and PF designed the Real Time PCR primers, performed the amplifications and melting curve analyses and participated in the revision of the manuscript, AC contributed to the revision of the draft manuscript for important intellectual content, SM conceived the study and thoroughly revised the manuscript. Finally, all the authors approved the manuscript to be published.

### Conflict of interest statement

The authors declare that the research was conducted in the absence of any commercial or financial relationships that could be construed as a potential conflict of interest.
