# Supplementary material for: Development of a High Resolution Virulence Allelic Profiling (HReVAP) Approach Based on the Accessory Genome of Escherichia coli to Characterize Shiga-Toxin Producing E. coli (STEC)
Source: Front Microbiol. 2016 Feb 23;7:202. doi: 10.3389/fmicb.2016.00202 (PMC4763077; doi:10.3389/fmicb.2016.00202)
Supplement: Supplementary file 2 [file Table2.docx]

***Supplementary Material***

**Development of a High Resolution Virulence Allelic Profiling (HReVAP) Approach Based on the Accessory Genome of *Escherichia coli* to Characterize Shiga-toxin Producing *E. coli* (STEC)**

**Valeria Michelacci^*^, Massimiliano Orsini, Arnold Knijn, Sabine Delannoy, Patrick Fach, Alfredo Caprioli and Stefano Morabito**

*** Correspondence:** Valeria Michelacci, European Reference Laboratory for Escherichia coli, Istituto Superiore di Sanità, Dipartimento di Sanità Pubblica Veterinaria e Sicurezza Alimentare, Viale Regina Elena 299, Rome 00161, Italy

valeria.michelacci@iss.it

**Supplementary Table 2. Intervals of Melting Temperature (Tm) defining the alleles identified for each Open Reading Frame (ORF).**

| ORF Id |  |  |  |  |  |  |  |  |  |  |  |  | Number of alleles | % of positive strains |
| --- | --- | --- | --- | --- | --- | --- | --- | --- | --- | --- | --- | --- | --- | --- |
| Z5101 | **Start of Tm interval** | 0 | 84,2054 | 84,72449 | 85,46979 | 86,45284 | 87,27985 | 87,6193 | 88,1646 |  |  |  |  |  |
|  | **Allele Id** | 98 | 1 | 2 | 3 | 4 | 5 | 6 | 99 |  |  |  | 6 | 29,313 |
|  | **Positive strains** | 0 | 12 | 167 | 21 | 5 | 1 | 3 | 0 |  |  |  |  |  |
| Z5102 | **Start of Tm interval** | 0 | 73,3746 | 73,89346 | 74,50744 | 75,0854 |  |  |  |  |  |  |  |  |
|  | **Allele Id** | 98 | 1 | 2 | 3 | 99 |  |  |  |  |  |  | 4 | 67,882 |
|  | **Positive strains** | 0 | 47 | 334 | 102 | 1 |  |  |  |  |  |  |  |  |
| Z5103 | **Start of Tm interval** | 0 | 76,88839 | 77,51971 | 78,10466 | 78,65509 | 79,22161 |  |  |  |  |  |  |  |
|  | **Allele Id** | 98 | 1 | 2 | 3 | 4 | 99 |  |  |  |  |  | 4 | 68,022 |
|  | **Positive strains** | 0 | 79 | 209 | 171 | 26 | 0 |  |  |  |  |  |  |  |
| Z5104 | **Start of Tm interval** | 0 | 78,19385 | 78,74435 | 79,29638 | 79,60582 | 80,00615 |  |  |  |  |  |  |  |
|  | **Allele Id** | 98 | 1 | 2 | 3 | 4 | 99 |  |  |  |  |  | 5 | 68,163 |
|  | **Positive strains** | 0 | 54 | 317 | 88 | 26 | 1 |  |  |  |  |  |  |  |
| Z5105 | **Start of Tm interval** | 0 | 80,05 | 80,53509 | 80,8901 | 81,52906 | 82,13741 | 82,76121 | 83,35 |  |  |  |  |  |
|  | **Allele Id** | 98 | 1 | 2 | 3 | 4 | 5 | 6 | 99 |  |  |  | 7 | 67,742 |
|  | **Positive strains** | 1 | 84 | 103 | 169 | 14 | 97 | 15 | 0 |  |  |  |  |  |
| Z5106 | **Start of Tm interval** | 0 | 80,99 | 81,48784 | 82,02471 | 82,58216 | 83,29203 | 84,09297 | 84,8701 | 85,48 |  |  |  |  |
|  | **Allele Id** | 98 | 1 | 2 | 3 | 4 | 5 | 6 | 7 | 99 |  |  | 8 | 68,022 |
|  | **Positive strains** | 0 | 1 | 19 | 86 | 77 | 49 | 228 | 25 | 1 |  |  |  |  |
| Z5107 | **Start of Tm interval** | 0 | 80,07863 | 80,46855 | 81,07276 | 81,64137 |  |  |  |  |  |  |  |  |
|  | **Allele Id** | 98 | 1 | 2 | 3 | 99 |  |  |  |  |  |  | 4 | 16,971 |
|  | **Positive strains** | 0 | 5 | 105 | 11 | 0 |  |  |  |  |  |  |  |  |
| Z5108 | **Start of Tm interval** | 0 | 75,9202 | 76,4629 | 76,97499 | 77,48784 | 78,1398 |  |  |  |  |  |  |  |
|  | **Allele Id** | 98 | 1 | 2 | 3 | 4 | 99 |  |  |  |  |  | 5 | 67,882 |
|  | **Positive strains** | 0 | 62 | 229 | 56 | 129 | 8 |  |  |  |  |  |  |  |
| Z5109 | **Start of Tm interval** | 0 | 77,3893 | 78,0919 | 78,80542 | 79,41436 | 79,9307 |  |  |  |  |  |  |  |
|  | **Allele Id** | 98 | 1 | 2 | 3 | 4 | 99 |  |  |  |  |  | 4 | 68,022 |
|  | **Positive strains** | 0 | 41 | 172 | 248 | 24 | 0 |  |  |  |  |  |  |  |
| Z5110 | **Start of Tm interval** | 0 | 76,98148 | 77,46433 | 78,01093 | 78,61212 | 79,13852 |  |  |  |  |  |  |  |
|  | **Allele Id** | 98 | 1 | 2 | 3 | 4 | 99 |  |  |  |  |  | 4 | 67,461 |
|  | **Positive strains** | 0 | 5 | 134 | 308 | 34 | 0 |  |  |  |  |  |  |  |
| Z5111 | **Start of Tm interval** | 0 | 80,14141 | 80,64953 | 81,21422 | 81,83363 | 82,35859 |  |  |  |  |  |  |  |
|  | **Allele Id** | 98 | 1 | 2 | 3 | 4 | 99 |  |  |  |  |  | 5 | 42,496 |
|  | **Positive strains** | 1 | 34 | 139 | 112 | 17 | 0 |  |  |  |  |  |  |  |
| Z5112 | **Start of Tm interval** | 0 | 79,43734 | 79,87179 | 80,45682 | 82,39029 | 84,35266 |  |  |  |  |  |  |  |
|  | **Allele Id** | 98 | 1 | 2 | 3 | 4 | 99 |  |  |  |  |  | 4 | 18,093 |
|  | **Positive strains** | 0 | 5 | 107 | 15 | 2 | 0 |  |  |  |  |  |  |  |
| Z5114 | **Start of Tm interval** | 0 | 76,98674 | 77,814 | 78,67326 |  |  |  |  |  |  |  |  |  |
|  | **Allele Id** | 98 | 1 | 2 | 99 |  |  |  |  |  |  |  | 2 | 16,55 |
|  | **Positive strains** | 0 | 9 | 109 | 0 |  |  |  |  |  |  |  |  |  |
| Z5116 | **Start of Tm interval** | 0 | 76,96 | 77,59306 | 78,35189 | 79,03158 | 79,6 |  |  |  |  |  |  |  |
|  | **Allele Id** | 98 | 1 | 2 | 3 | 4 | 99 |  |  |  |  |  | 5 | 68,163 |
|  | **Positive strains** | 0 | 5 | 126 | 271 | 83 | 1 |  |  |  |  |  |  |  |
| Z5117 | **Start of Tm interval** | 0 | 78,11183 | 78,6442 | 79,22201 | 79,69817 |  |  |  |  |  |  |  |  |
|  | **Allele Id** | 98 | 1 | 2 | 3 | 99 |  |  |  |  |  |  | 4 | 43,058 |
|  | **Positive strains** | 1 | 44 | 239 | 23 | 0 |  |  |  |  |  |  |  |  |
| Z5118 | **Start of Tm interval** | 0 | 76,77816 | 77,14133 | 77,41138 | 77,8127 | 78,37281 | 78,99184 |  |  |  |  |  |  |
|  | **Allele Id** | 98 | 1 | 2 | 3 | 4 | 5 | 99 |  |  |  |  | 6 | 68,022 |
|  | **Positive strains** | 0 | 7 | 32 | 145 | 162 | 128 | 11 |  |  |  |  |  |  |
| Z5119 | **Start of Tm interval** | 0 | 79,14 | 79,55252 | 80,07427 | 80,60902 | 81,1083 | 81,62 |  |  |  |  |  |  |
|  | **Allele Id** | 98 | 1 | 2 | 3 | 4 | 5 | 99 |  |  |  |  | 6 | 53,576 |
|  | **Positive strains** | 0 | 6 | 178 | 147 | 38 | 12 | 1 |  |  |  |  |  |  |
| Z5120 | **Start of Tm interval** | 0 | 79,78564 | 80,19029 | 80,685 | 81,23436 |  |  |  |  |  |  |  |  |
|  | **Allele Id** | 98 | 1 | 2 | 3 | 99 |  |  |  |  |  |  | 3 | 67,742 |
|  | **Positive strains** | 0 | 75 | 350 | 58 | 0 |  |  |  |  |  |  |  |  |
| Z5121 | **Start of Tm interval** | 0 | 76,4811 | 76,96262 | 77,37415 | 78,10249 | 78,7189 |  |  |  |  |  |  |  |
|  | **Allele Id** | 98 | 1 | 2 | 3 | 4 | 99 |  |  |  |  |  | 4 | 45,722 |
|  | **Positive strains** | 0 | 23 | 34 | 244 | 25 | 0 |  |  |  |  |  |  |  |
| Z5122 | **Start of Tm interval** | 0 | 82,45307 | 82,85494 | 83,41817 | 83,88693 |  |  |  |  |  |  |  |  |
|  | **Allele Id** | 98 | 1 | 2 | 3 | 99 |  |  |  |  |  |  | 3 | 16,971 |
|  | **Positive strains** | 0 | 6 | 99 | 16 | 0 |  |  |  |  |  |  |  |  |
| Z5123 | **Start of Tm interval** | 0 | 80,3053 | 80,91159 | 81,5554 | 82,22161 | 82,68134 | 83,1047 |  |  |  |  |  |  |
|  | **Allele Id** | 98 | 1 | 2 | 3 | 4 | 5 | 99 |  |  |  |  | 5 | 48,948 |
|  | **Positive strains** | 0 | 40 | 120 | 145 | 34 | 10 | 0 |  |  |  |  |  |  |
| Z5124 | **Start of Tm interval** | 0 | 75,5562 | 76,14537 | 76,87182 | 77,54971 | 78,1138 |  |  |  |  |  |  |  |
|  | **Allele Id** | 98 | 1 | 2 | 3 | 4 | 99 |  |  |  |  |  | 5 | 67,742 |
|  | **Positive strains** | 0 | 39 | 147 | 139 | 152 | 6 |  |  |  |  |  |  |  |
| Z5125 | **Start of Tm interval** | 0 | 77,3248 | 77,91126 | 78,44882 | 78,98231 | 79,51933 | 80,0752 |  |  |  |  |  |  |
|  | **Allele Id** | 98 | 1 | 2 | 3 | 4 | 5 | 99 |  |  |  |  | 6 | 61,571 |
|  | **Positive strains** | 0 | 8 | 146 | 148 | 72 | 60 | 5 |  |  |  |  |  |  |
| Z5126 | **Start of Tm interval** | 0 | 79,02013 | 79,49095 | 79,8647 | 80,25672 | 80,76648 | 81,23987 |  |  |  |  |  |  |
|  | **Allele Id** | 98 | 1 | 2 | 3 | 4 | 5 | 99 |  |  |  |  | 5 | 68,022 |
|  | **Positive strains** | 0 | 40 | 88 | 102 | 229 | 26 | 0 |  |  |  |  |  |  |
| Z5127 | **Start of Tm interval** | 0 | 77,80579 | 78,14647 | 78,43669 | 78,94831 | 79,35421 |  |  |  |  |  |  |  |
|  | **Allele Id** | 98 | 1 | 2 | 3 | 4 | 99 |  |  |  |  |  | 4 | 43,759 |
|  | **Positive strains** | 0 | 35 | 11 | 241 | 25 | 0 |  |  |  |  |  |  |  |
| Z5128 | **Start of Tm interval** | 0 | 72,0898 | 72,618 | 73,13783 | 73,63896 | 74,1202 |  |  |  |  |  |  |  |
|  | **Allele Id** | 98 | 1 | 2 | 3 | 4 | 99 |  |  |  |  |  | 4 | 67,742 |
|  | **Positive strains** | 0 | 42 | 294 | 139 | 8 | 0 |  |  |  |  |  |  |  |
| Z5129 | **Start of Tm interval** | 0 | 74,88686 | 75,37035 | 75,90284 | 76,36769 | 76,78314 |  |  |  |  |  |  |  |
|  | **Allele Id** | 98 | 1 | 2 | 3 | 4 | 99 |  |  |  |  |  | 5 | 67,602 |
|  | **Positive strains** | 0 | 1 | 58 | 309 | 107 | 7 |  |  |  |  |  |  |  |
| Z5131 | **Start of Tm interval** | 0 | 76,01192 | 76,42972 | 76,94916 | 77,48399 | 77,9127 | 78,2447 | 78,76079 | 79,21808 |  |  |  |  |
|  | **Allele Id** | 98 | 1 | 2 | 3 | 4 | 5 | 6 | 7 | 99 |  |  | 7 | 67,742 |
|  | **Positive strains** | 0 | 2 | 52 | 207 | 71 | 14 | 121 | 16 | 0 |  |  |  |  |
| Z5132 | **Start of Tm interval** | 0 | 76,0894 | 76,6357 | 77,22241 | 77,8338 | 78,3206 |  |  |  |  |  |  |  |
|  | **Allele Id** | 98 | 1 | 2 | 3 | 4 | 99 |  |  |  |  |  | 5 | 60,729 |
|  | **Positive strains** | 2 | 118 | 182 | 112 | 19 | 0 |  |  |  |  |  |  |  |
| Z5133 | **Start of Tm interval** | 0 | 73,4817 | 74,00027 | 74,57012 | 75,07237 | 75,5683 |  |  |  |  |  |  |  |
|  | **Allele Id** | 98 | 1 | 2 | 3 | 4 | 99 |  |  |  |  |  | 4 | 67,882 |
|  | **Positive strains** | 0 | 30 | 289 | 156 | 9 | 0 |  |  |  |  |  |  |  |
| Z5134 | **Start of Tm interval** | 0 | 77,9684 | 78,66789 | 79,42038 | 79,93642 | 80,54504 | 81,1516 |  |  |  |  |  |  |
|  | **Allele Id** | 98 | 1 | 2 | 3 | 4 | 5 | 99 |  |  |  |  | 5 | 66,199 |
|  | **Positive strains** | 0 | 36 | 251 | 55 | 120 | 10 | 0 |  |  |  |  |  |  |
| Z5135 | **Start of Tm interval** | 0 | 74,90408 | 75,39148 | 75,99891 | 76,61911 | 77,11592 |  |  |  |  |  |  |  |
|  | **Allele Id** | 98 | 1 | 2 | 3 | 4 | 99 |  |  |  |  |  | 4 | 67,602 |
|  | **Positive strains** | 0 | 6 | 167 | 292 | 17 | 0 |  |  |  |  |  |  |  |
| Z5136 | **Start of Tm interval** | 0 | 74,04931 | 74,60021 | 75,15829 | 75,6241 | 76,12069 |  |  |  |  |  |  |  |
|  | **Allele Id** | 98 | 1 | 2 | 3 | 4 | 99 |  |  |  |  |  | 4 | 64,236 |
|  | **Positive strains** | 0 | 45 | 286 | 115 | 12 | 0 |  |  |  |  |  |  |  |
| Z5137 | **Start of Tm interval** | 0 | 75,87 | 76,36354 | 76,82976 | 77,247 | 77,69 |  |  |  |  |  |  |  |
|  | **Allele Id** | 98 | 1 | 2 | 3 | 4 | 99 |  |  |  |  |  | 4 | 68,443 |
|  | **Positive strains** | 0 | 77 | 335 | 74 | 2 | 0 |  |  |  |  |  |  |  |
| Z5139 | **Start of Tm interval** | 0 | 74,23134 | 74,74347 | 75,24451 | 75,6541 | 76,08866 |  |  |  |  |  |  |  |
|  | **Allele Id** | 98 | 1 | 2 | 3 | 4 | 99 |  |  |  |  |  | 4 | 68,303 |
|  | **Positive strains** | 0 | 87 | 343 | 53 | 4 | 0 |  |  |  |  |  |  |  |
| Z5140 | **Start of Tm interval** | 0 | 79,50773 | 80,14843 | 80,84024 | 81,39571 | 81,93284 | 82,34227 |  |  |  |  |  |  |
|  | **Allele Id** | 98 | 1 | 2 | 3 | 4 | 5 | 99 |  |  |  |  | 5 | 61,571 |
|  | **Positive strains** | 0 | 1 | 82 | 139 | 198 | 19 | 0 |  |  |  |  |  |  |
| Z5142 | **Start of Tm interval** | 0 | 79,0574 | 79,56227 | 80,19848 | 80,72681 | 81,15621 | 81,5926 |  |  |  |  |  |  |
|  | **Allele Id** | 98 | 1 | 2 | 3 | 4 | 5 | 99 |  |  |  |  | 5 | 67,882 |
|  | **Positive strains** | 0 | 37 | 268 | 112 | 63 | 4 | 0 |  |  |  |  |  |  |
| Z5143 | **Start of Tm interval** | 0 | 77,0471 | 78,58209 | 80,049 | 80,60983 | 81,72194 | 82,92659 | 83,59551 | 84,1329 |  |  |  |  |
|  | **Allele Id** | 98 | 1 | 2 | 3 | 4 | 5 | 6 | 7 | 99 |  |  | 8 | 67,882 |
|  | **Positive strains** | 0 | 4 | 47 | 220 | 36 | 40 | 124 | 13 | 1 |  |  |  |  |
| Z4318 | **Start of Tm interval** | 0 | 77,35132 | 77,9604 | 78,6062 | 79,03868 |  |  |  |  |  |  |  |  |
|  | **Allele Id** | 98 | 1 | 2 | 3 | 99 |  |  |  |  |  |  | 4 | 34,502 |
|  | **Positive strains** | 0 | 46 | 178 | 21 | 1 |  |  |  |  |  |  |  |  |
| Z4320 | **Start of Tm interval** | 0 | 78,3144 | 78,96937 | 79,64166 | 80,18393 | 80,6956 |  |  |  |  |  |  |  |
|  | **Allele Id** | 98 | 1 | 2 | 3 | 4 | 99 |  |  |  |  |  | 4 | 45,302 |
|  | **Positive strains** | 0 | 50 | 184 | 79 | 10 | 0 |  |  |  |  |  |  |  |
| Z4321 | **Start of Tm interval** | 0 | 75,1014 | 75,58684 | 76,08843 | 76,5086 |  |  |  |  |  |  |  |  |
|  | **Allele Id** | 98 | 1 | 2 | 3 | 99 |  |  |  |  |  |  | 4 | 40,673 |
|  | **Positive strains** | 0 | 55 | 210 | 24 | 1 |  |  |  |  |  |  |  |  |
| Z4322 | **Start of Tm interval** | 0 | 85,22574 | 85,59513 | 85,96721 | 86,56658 | 87,01426 |  |  |  |  |  |  |  |
|  | **Allele Id** | 98 | 1 | 2 | 3 | 4 | 99 |  |  |  |  |  | 4 | 33,801 |
|  | **Positive strains** | 0 | 38 | 11 | 164 | 28 | 0 |  |  |  |  |  |  |  |
| Z4325 | **Start of Tm interval** | 0 | 77,94328 | 78,24327 | 78,52615 | 78,77714 | 79,16131 | 79,42789 | 79,89686 | 80,40672 |  |  |  |  |
|  | **Allele Id** | 98 | 1 | 2 | 3 | 4 | 5 | 6 | 7 | 99 |  |  | 8 | 51,613 |
|  | **Positive strains** | 0 | 22 | 48 | 17 | 217 | 46 | 16 | 2 | 1 |  |  |  |  |
| Z4326 | **Start of Tm interval** | 0 | 75,03778 | 75,62276 | 76,22973 | 76,78126 | 77,22222 |  |  |  |  |  |  |  |
|  | **Allele Id** | 98 | 1 | 2 | 3 | 4 | 99 |  |  |  |  |  | 4 | 45,582 |
|  | **Positive strains** | 0 | 38 | 111 | 145 | 31 | 0 |  |  |  |  |  |  |  |
| Z4327 | **Start of Tm interval** | 0 | 72,1217 | 72,49686 | 72,96563 | 73,54763 | 74,0783 |  |  |  |  |  |  |  |
|  | **Allele Id** | 98 | 1 | 2 | 3 | 4 | 99 |  |  |  |  |  | 4 | 51,613 |
|  | **Positive strains** | 0 | 2 | 88 | 236 | 42 | 0 |  |  |  |  |  |  |  |
| Z4328 | **Start of Tm interval** | 0 | 77,18151 | 77,73225 | 78,29834 | 78,71849 |  |  |  |  |  |  |  |  |
|  | **Allele Id** | 98 | 1 | 2 | 3 | 99 |  |  |  |  |  |  | 3 | 45,582 |
|  | **Positive strains** | 0 | 83 | 215 | 27 | 0 |  |  |  |  |  |  |  |  |
| Z4329 | **Start of Tm interval** | 0 | 77,54283 | 77,9751 | 78,59591 | 79,178 | 79,58717 |  |  |  |  |  |  |  |
|  | **Allele Id** | 98 | 1 | 2 | 3 | 4 | 99 |  |  |  |  |  | 4 | 42,777 |
|  | **Positive strains** | 0 | 1 | 79 | 208 | 17 | 0 |  |  |  |  |  |  |  |
| Z4331 | **Start of Tm interval** | 0 | 80,02185 | 80,47782 | 80,94139 | 81,50509 | 81,99815 |  |  |  |  |  |  |  |
|  | **Allele Id** | 98 | 1 | 2 | 3 | 4 | 99 |  |  |  |  |  | 4 | 37,868 |
|  | **Positive strains** | 0 | 26 | 46 | 180 | 18 | 0 |  |  |  |  |  |  |  |
| Z4332 | **Start of Tm interval** | 0 | 76,39463 | 76,83452 | 77,3384 | 77,71537 |  |  |  |  |  |  |  |  |
|  | **Allele Id** | 98 | 1 | 2 | 3 | 99 |  |  |  |  |  |  | 3 | 37,728 |
|  | **Positive strains** | 0 | 81 | 174 | 14 | 0 |  |  |  |  |  |  |  |  |
| Z4333 | **Start of Tm interval** | 0 | 77,04225 | 77,46196 | 77,74595 | 78,18227 | 78,55775 |  |  |  |  |  |  |  |
|  | **Allele Id** | 98 | 1 | 2 | 3 | 4 | 99 |  |  |  |  |  | 4 | 37,307 |
|  | **Positive strains** | 0 | 77 | 11 | 166 | 12 | 0 |  |  |  |  |  |  |  |
| Z2036 | **Start of Tm interval** | 0 | 80,2079 | 80,667 | 81,1981 | 81,686 | 82,1164 | 82,572 |  |  |  |  |  |  |
|  | **Allele Id** | 98 | 1 | 2 | 3 | 4 | 5 | 99 |  |  |  |  | 5 | 46,424 |
|  | **Positive strains** | 0 | 82 | 206 | 31 | 8 | 4 | 0 |  |  |  |  |  |  |
| Z2037 | **Start of Tm interval** | 0 | 81,45 | 81,965 | 82,4066 | 82,813 | 83,35 |  |  |  |  |  |  |  |
|  | **Allele Id** | 98 | 1 | 2 | 3 | 4 | 99 |  |  |  |  |  | 6 | 78,261 |
|  | **Positive strains** | 3 | 39 | 223 | 195 | 92 | 6 |  |  |  |  |  |  |  |
| Z2039 | **Start of Tm interval** | 0 | 79,0979 | 79,637 | 80,2068 | 80,652 |  |  |  |  |  |  |  |  |
|  | **Allele Id** | 98 | 1 | 2 | 3 | 99 |  |  |  |  |  |  | 5 | 81,066 |
|  | **Positive strains** | 1 | 107 | 430 | 38 | 2 |  |  |  |  |  |  |  |  |
| Z2045 | **Start of Tm interval** | 0 | 82,3156 | 82,75 | 83,1962 | 83,483 | 83,8736 | 84,314 |  |  |  |  |  |  |
|  | **Allele Id** | 98 | 1 | 2 | 3 | 4 | 5 | 99 |  |  |  |  | 5 | 40,954 |
|  | **Positive strains** | 0 | 4 | 73 | 86 | 111 | 18 | 0 |  |  |  |  |  |  |
| Z2046 | **Start of Tm interval** | 0 | 84,861 | 85,194 | 85,5907 | 85,989 | 86,4434 | 86,782 | 87,2798 | 87,739 |  |  |  |  |
|  | **Allele Id** | 98 | 1 | 2 | 3 | 4 | 5 | 6 | 7 | 99 |  |  | 8 | 49,229 |
|  | **Positive strains** | 1 | 6 | 29 | 21 | 103 | 38 | 141 | 12 | 0 |  |  |  |  |
| Z2048 | **Start of Tm interval** | 0 | 83,0754 | 83,588 | 84,0374 | 84,517 | 85,1179 | 85,635 |  |  |  |  |  |  |
|  | **Allele Id** | 98 | 1 | 2 | 3 | 4 | 5 | 99 |  |  |  |  | 6 | 43,058 |
|  | **Positive strains** | 0 | 3 | 11 | 80 | 184 | 27 | 2 |  |  |  |  |  |  |
| Z2053 | **Start of Tm interval** | 0 | 82,4447 | 82,815 | 83,2155 | 83,513 | 83,9422 | 84,405 |  |  |  |  |  |  |
|  | **Allele Id** | 98 | 1 | 2 | 3 | 4 | 5 | 99 |  |  |  |  | 6 | 44,039 |
|  | **Positive strains** | 0 | 7 | 69 | 81 | 138 | 18 | 1 |  |  |  |  |  |  |
| Z2054 | **Start of Tm interval** | 0 | 79,48 | 79,951 | 80,3492 | 80,844 | 81,3559 | 81,83 |  |  |  |  |  |  |
|  | **Allele Id** | 98 | 1 | 2 | 3 | 4 | 5 | 99 |  |  |  |  | 6 | 99,439 |
|  | **Positive strains** | 0 | 4 | 38 | 238 | 382 | 45 | 2 |  |  |  |  |  |  |
| Z2056 | **Start of Tm interval** | 0 | 81,8461 | 82,393 | 82,9934 | 83,394 | 83,7416 | 84,21 | 84,6739 |  |  |  |  |  |
|  | **Allele Id** | 98 | 1 | 2 | 3 | 4 | 5 | 6 | 99 |  |  |  | 7 | 89,481 |
|  | **Positive strains** | 2 | 92 | 390 | 111 | 24 | 15 | 4 | 0 |  |  |  |  |  |
| Z2057 | **Start of Tm interval** | 0 | 82,66 | 83,145 | 83,5026 | 83,829 | 84,2244 | 84,953 | 85,5349 | 85,822 | 86,5649 | 87,39 |  |  |
|  | **Allele Id** | 98 | 1 | 2 | 3 | 4 | 5 | 6 | 7 | 8 | 9 | 99 | 9 | 66,059 |
|  | **Positive strains** | 0 | 7 | 16 | 27 | 40 | 190 | 147 | 44 | 51 | 13 | 0 |  |  |
| Z2060 | **Start of Tm interval** | 0 | 83,3407 | 83,845 | 84,3856 | 84,956 | 85,629 | 86,259 |  |  |  |  |  |  |
|  | **Allele Id** | 98 | 1 | 2 | 3 | 4 | 5 | 99 |  |  |  |  | 5 | 50,771 |
|  | **Positive strains** | 0 | 27 | 99 | 89 | 137 | 10 | 0 |  |  |  |  |  |  |
| Z2065 | **Start of Tm interval** | 0 | 81,5335 | 81,847 | 82,1455 | 82,434 | 82,8035 | 83,186 |  |  |  |  |  |  |
|  | **Allele Id** | 98 | 1 | 2 | 3 | 4 | 5 | 99 |  |  |  |  | 5 | 48,107 |
|  | **Positive strains** | 0 | 29 | 62 | 119 | 106 | 27 | 0 |  |  |  |  |  |  |
| Z2066 | **Start of Tm interval** | 0 | 81,1375 | 81,527 | 81,8579 | 82,221 | 82,6025 |  |  |  |  |  |  |  |
|  | **Allele Id** | 98 | 1 | 2 | 3 | 4 | 99 |  |  |  |  |  | 4 | 68,303 |
|  | **Positive strains** | 0 | 82 | 214 | 170 | 21 | 0 |  |  |  |  |  |  |  |
| Z2069 | **Start of Tm interval** | 0 | 84,2 | 84,772 | 85,1904 | 85,491 | 85,7931 | 86,108 | 86,3732 | 87,041 | 87,674 | 88,15 |  |  |
|  | **Allele Id** | 98 | 1 | 2 | 3 | 4 | 5 | 6 | 7 | 8 | 9 | 99 | 9 | 84,572 |
|  | **Positive strains** | 0 | 41 | 108 | 107 | 89 | 85 | 51 | 122 | 37 | 3 | 0 |  |  |
| Z2071 | **Start of Tm interval** | 0 | 80,957 | 81,418 | 81,9523 | 82,519 | 82,9211 | 83,42 | 83,933 |  |  |  |  |  |
|  | **Allele Id** | 98 | 1 | 2 | 3 | 4 | 5 | 6 | 99 |  |  |  | 6 | 87,377 |
|  | **Positive strains** | 0 | 5 | 109 | 374 | 91 | 40 | 4 | 0 |  |  |  |  |  |
| Z2073 | **Start of Tm interval** | 0 | 84,4107 | 85,062 | 85,7117 | 86,281 | 86,8639 | 87,469 |  |  |  |  |  |  |
|  | **Allele Id** | 98 | 1 | 2 | 3 | 4 | 5 | 99 |  |  |  |  | 6 | 73,773 |
|  | **Positive strains** | 0 | 13 | 143 | 290 | 73 | 6 | 1 |  |  |  |  |  |  |
| Z2082 | **Start of Tm interval** | 0 | 82,5411 | 83,005 | 83,5118 | 83,929 | 84,3796 | 84,849 |  |  |  |  |  |  |
|  | **Allele Id** | 98 | 1 | 2 | 3 | 4 | 5 | 99 |  |  |  |  | 5 | 29,593 |
|  | **Positive strains** | 0 | 27 | 120 | 42 | 21 | 1 | 0 |  |  |  |  |  |  |
| Z2084 | **Start of Tm interval** | 0 | 78,21 | 78,832 | 79,6755 | 80,418 | 80,95 | 81,38 |  |  |  |  |  |  |
|  | **Allele Id** | 98 | 1 | 2 | 3 | 4 | 5 | 99 |  |  |  |  | 6 | 73,352 |
|  | **Positive strains** | 0 | 49 | 304 | 81 | 72 | 16 | 1 |  |  |  |  |  |  |
| Z2085 | **Start of Tm interval** | 0 | 81,1115 | 81,508 | 81,902 | 82,494 | 83,0524 | 83,377 | 85,5186 | 87,769 |  |  |  |  |
|  | **Allele Id** | 98 | 1 | 2 | 3 | 4 | 5 | 6 | 7 | 99 |  |  | 8 | 38,85 |
|  | **Positive strains** | 1 | 12 | 82 | 131 | 31 | 4 | 4 | 12 | 0 |  |  |  |  |
| Z2086 | **Start of Tm interval** | 0 | 78,815 | 79,17 | 79,4575 | 79,735 | 80,0601 | 80,435 |  |  |  |  |  |  |
|  | **Allele Id** | 98 | 1 | 2 | 3 | 4 | 5 | 99 |  |  |  |  | 6 | 64,516 |
|  | **Positive strains** | 0 | 24 | 109 | 110 | 135 | 72 | 10 |  |  |  |  |  |  |
| Z2090 | **Start of Tm interval** | 0 | 81,3159 | 81,833 | 82,3315 | 82,697 | 83,1342 |  |  |  |  |  |  |  |
|  | **Allele Id** | 98 | 1 | 2 | 3 | 4 | 99 |  |  |  |  |  | 4 | 21,879 |
|  | **Positive strains** | 0 | 13 | 105 | 24 | 14 | 0 |  |  |  |  |  |  |  |
| Z2091 | **Start of Tm interval** | 0 | 80,9052 | 81,187 | 81,4375 | 81,704 | 81,9683 | 82,315 |  |  |  |  |  |  |
|  | **Allele Id** | 98 | 1 | 2 | 3 | 4 | 5 | 99 |  |  |  |  | 5 | 21,459 |
|  | **Positive strains** | 0 | 14 | 49 | 57 | 19 | 14 | 0 |  |  |  |  |  |  |
| Z2093 | **Start of Tm interval** | 0 | 81,378 | 81,911 | 82,4992 | 83,104 | 83,662 |  |  |  |  |  |  |  |
|  | **Allele Id** | 98 | 1 | 2 | 3 | 4 | 99 |  |  |  |  |  | 5 | 52,454 |
|  | **Positive strains** | 0 | 41 | 128 | 133 | 71 | 1 |  |  |  |  |  |  |  |
| Z2094 | **Start of Tm interval** | 0 | 80,1179 | 80,467 | 80,8901 | 81,509 | 82,1026 | 82,467 | 82,8321 |  |  |  |  |  |
|  | **Allele Id** | 98 | 1 | 2 | 3 | 4 | 5 | 6 | 99 |  |  |  | 6 | 63,815 |
|  | **Positive strains** | 0 | 9 | 56 | 241 | 111 | 27 | 11 | 0 |  |  |  |  |  |
| Z2096 | **Start of Tm interval** | 0 | 82,1464 | 82,734 | 83,4653 | 84,203 | 84,8106 | 85,397 | 85,9136 |  |  |  |  |  |
|  | **Allele Id** | 98 | 1 | 2 | 3 | 4 | 5 | 6 | 99 |  |  |  | 7 | 67,321 |
|  | **Positive strains** | 0 | 4 | 72 | 272 | 89 | 40 | 2 | 1 |  |  |  |  |  |
| Z2100 | **Start of Tm interval** | 0 | 84,5051 | 84,823 | 85,2952 | 85,789 | 86,1245 | 86,505 |  |  |  |  |  |  |
|  | **Allele Id** | 98 | 1 | 2 | 3 | 4 | 5 | 99 |  |  |  |  | 5 | 33,38 |
|  | **Positive strains** | 0 | 4 | 63 | 129 | 30 | 12 | 0 |  |  |  |  |  |  |
| Z2101 | **Start of Tm interval** | 0 | 82,3861 | 82,837 | 83,3536 | 83,841 | 84,3834 | 84,897 | 85,3639 |  |  |  |  |  |
|  | **Allele Id** | 98 | 1 | 2 | 3 | 4 | 5 | 6 | 99 |  |  |  | 8 | 92,146 |
|  | **Positive strains** | 1 | 8 | 96 | 147 | 263 | 118 | 23 | 1 |  |  |  |  |  |
| Z2102 | **Start of Tm interval** | 0 | 80,88 | 81,333 | 81,8544 | 82,551 | 83,0698 | 83,508 | 83,9296 | 84,468 | 85,1 |  |  |  |
|  | **Allele Id** | 98 | 1 | 2 | 3 | 4 | 5 | 6 | 7 | 8 | 99 |  | 10 | 51,473 |
|  | **Positive strains** | 2 | 6 | 53 | 176 | 36 | 12 | 16 | 66 | 54 | 3 |  |  |  |
| Z2104 | **Start of Tm interval** | 0 | 78,9874 | 79,468 | 79,9549 | 80,437 | 80,9222 | 81,632 | 82,2826 |  |  |  |  |  |
|  | **Allele Id** | 98 | 1 | 2 | 3 | 4 | 5 | 6 | 99 |  |  |  | 7 | 54,137 |
|  | **Positive strains** | 0 | 14 | 44 | 101 | 57 | 123 | 46 | 1 |  |  |  |  |  |
| Z2108 | **Start of Tm interval** | 0 | 82,1547 | 82,736 | 83,3172 | 83,821 | 84,2534 | 84,745 |  |  |  |  |  |  |
|  | **Allele Id** | 98 | 1 | 2 | 3 | 4 | 5 | 99 |  |  |  |  | 5 | 72,23 |
|  | **Positive strains** | 0 | 3 | 108 | 269 | 105 | 30 | 0 |  |  |  |  |  |  |
| Z2109 | **Start of Tm interval** | 0 | 84,4043 | 84,869 | 85,2421 | 85,481 | 85,7426 | 86,049 | 86,4957 |  |  |  |  |  |
|  | **Allele Id** | 98 | 1 | 2 | 3 | 4 | 5 | 6 | 99 |  |  |  | 7 | 69,565 |
|  | **Positive strains** | 0 | 30 | 53 | 79 | 157 | 140 | 36 | 1 |  |  |  |  |  |
| Z2112 | **Start of Tm interval** | 0 | 84,101 | 84,464 | 84,9674 | 85,589 | 85,9844 | 86,319 | 86,709 |  |  |  |  |  |
|  | **Allele Id** | 98 | 1 | 2 | 3 | 4 | 5 | 6 | 99 |  |  |  | 7 | 40,252 |
|  | **Positive strains** | 0 | 2 | 36 | 192 | 38 | 15 | 3 | 1 |  |  |  |  |  |
| Z2114 | **Start of Tm interval** | 0 | 81,7766 | 82,192 | 82,7587 | 83,45 | 84,0334 |  |  |  |  |  |  |  |
|  | **Allele Id** | 98 | 1 | 2 | 3 | 4 | 99 |  |  |  |  |  | 4 | 39,551 |
|  | **Positive strains** | 0 | 2 | 52 | 213 | 15 | 0 |  |  |  |  |  |  |  |
| Z2116 | **Start of Tm interval** | 0 | 82,3882 | 82,937 | 83,4218 | 83,766 | 84,2018 |  |  |  |  |  |  |  |
|  | **Allele Id** | 98 | 1 | 2 | 3 | 4 | 99 |  |  |  |  |  | 6 | 40,813 |
|  | **Positive strains** | 1 | 46 | 160 | 49 | 33 | 2 |  |  |  |  |  |  |  |
| Z2118 | **Start of Tm interval** | 0 | 81,8949 | 82,59 | 83,3621 | 83,861 | 84,3051 |  |  |  |  |  |  |  |
|  | **Allele Id** | 98 | 1 | 2 | 3 | 4 | 99 |  |  |  |  |  | 5 | 86,816 |
|  | **Positive strains** | 0 | 68 | 396 | 116 | 36 | 3 |  |  |  |  |  |  |  |
| Z2120 | **Start of Tm interval** | 0 | 81,9754 | 82,397 | 82,7903 | 83,346 | 83,8751 | 84,385 | 84,8946 |  |  |  |  |  |
|  | **Allele Id** | 98 | 1 | 2 | 3 | 4 | 5 | 6 | 99 |  |  |  | 6 | 60,449 |
|  | **Positive strains** | 0 | 33 | 150 | 196 | 33 | 16 | 3 | 0 |  |  |  |  |  |
| Z2121 | **Start of Tm interval** | 0 | 76,8292 | 77,274 | 77,7633 | 78,35 | 78,8308 |  |  |  |  |  |  |  |
|  | **Allele Id** | 98 | 1 | 2 | 3 | 4 | 99 |  |  |  |  |  | 4 | 53,857 |
|  | **Positive strains** | 0 | 41 | 87 | 234 | 22 | 0 |  |  |  |  |  |  |  |
| Z2131 | **Start of Tm interval** | 0 | 82,33 | 82,842 | 83,4059 | 83,982 | 84,5 |  |  |  |  |  |  |  |
|  | **Allele Id** | 98 | 1 | 2 | 3 | 4 | 99 |  |  |  |  |  | 4 | 82,468 |
|  | **Positive strains** | 0 | 61 | 331 | 164 | 32 | 0 |  |  |  |  |  |  |  |
| Z2146 | **Start of Tm interval** | 0 | 84,3707 | 84,76 | 85,0833 | 85,566 | 86,2152 | 86,756 | 87,2113 | 87,779 |  |  |  |  |
|  | **Allele Id** | 98 | 1 | 2 | 3 | 4 | 5 | 6 | 7 | 99 |  |  | 8 | 93,689 |
|  | **Positive strains** | 1 | 4 | 19 | 101 | 434 | 99 | 7 | 3 | 0 |  |  |  |  |
| Z2150 | **Start of Tm interval** | 0 | 81,38 | 81,946 | 82,4867 | 83 |  |  |  |  |  |  |  |  |
|  | **Allele Id** | 98 | 1 | 2 | 3 | 99 |  |  |  |  |  |  | 3 | 41,374 |
|  | **Positive strains** | 0 | 127 | 146 | 22 | 0 |  |  |  |  |  |  |  |  |
| Z2152 | **Start of Tm interval** | 0 | 79,97 | 80,446 | 80,4299 | 80,935 | 81,6758 | 82,22 |  |  |  |  |  |  |
|  | **Allele Id** | 98 | 1 | 2 | 3 | 4 | 5 | 99 |  |  |  |  | 5 | 34,642 |
|  | **Positive strains** | 0 | 3 | 111 | 104 | 28 | 1 | 0 |  |  |  |  |  |  |

**Supplementary Table 3. Number of alleles identified for each of the ORFs assayed.**

| **Pathogenicity island** | **ORF identifier** | **Number of alleles** |  |
| --- | --- | --- | --- |
| LEE | Z5101 | 6 | **Mean=4.79**  **Range=2 - 8**  **Median=5** |
|  | Z5102 | 4 |  |
|  | Z5103 | 4 |  |
|  | Z5104 | 5 |  |
|  | Z5105 | 7 |  |
|  | Z5106 | 8 |  |
|  | Z5107 | 4 |  |
|  | Z5108 | 5 |  |
|  | Z5109 | 4 |  |
|  | Z5110 | 4 |  |
|  | Z5111 | 5 |  |
|  | Z5112 | 4 |  |
|  | Z5114 | 2 |  |
|  | Z5116 | 5 |  |
|  | Z5117 | 4 |  |
|  | Z5118 | 6 |  |
|  | Z5119 | 6 |  |
|  | Z5120 | 3 |  |
|  | Z5121 | 4 |  |
|  | Z5122 | 3 |  |
|  | Z5123 | 5 |  |
|  | Z5124 | 5 |  |
|  | Z5125 | 6 |  |
|  | Z5126 | 5 |  |
|  | Z5127 | 4 |  |
|  | Z5128 | 4 |  |
|  | Z5129 | 5 |  |
|  | Z5131 | 7 |  |
|  | Z5132 | 5 |  |
|  | Z5133 | 4 |  |
|  | Z5134 | 5 |  |
|  | Z5135 | 4 |  |
|  | Z5136 | 4 |  |
|  | Z5137 | 4 |  |
|  | Z5139 | 4 |  |
|  | Z5140 | 5 |  |
|  | Z5142 | 5 |  |
|  | Z5143 | 8 |  |
| OI-122 | Z4318 | 4 | **Mean=4.17**  **Range=3 - 8**  **Median=4** |
|  | Z4320 | 4 |  |
|  | Z4321 | 4 |  |
|  | Z4322 | 4 |  |
|  | Z4325 | 8 |  |
|  | Z4326 | 4 |  |
|  | Z4327 | 4 |  |
|  | Z4328 | 3 |  |
|  | Z4329 | 4 |  |
|  | Z4331 | 4 |  |
|  | Z4332 | 3 |  |
|  | Z4333 | 4 |  |
| OI-57 | Z2036 | 5 | **Mean=5.95**  **Range=3 - 10**  **Median=6** |
|  | Z2037 | 6 |  |
|  | Z2039 | 5 |  |
|  | Z2045 | 5 |  |
|  | Z2046 | 8 |  |
|  | Z2048 | 6 |  |
|  | Z2053 | 6 |  |
|  | Z2054 | 6 |  |
|  | Z2056 | 7 |  |
|  | Z2057 | 9 |  |
|  | Z2060 | 5 |  |
|  | Z2065 | 5 |  |
|  | Z2066 | 4 |  |
|  | Z2069 | 9 |  |
|  | Z2071 | 6 |  |
|  | Z2073 | 6 |  |
|  | Z2082 | 5 |  |
|  | Z2084 | 6 |  |
|  | Z2085 | 8 |  |
|  | Z2086 | 6 |  |
|  | Z2090 | 4 |  |
|  | Z2091 | 5 |  |
|  | Z2093 | 5 |  |
|  | Z2094 | 6 |  |
|  | Z2096 | 7 |  |
|  | Z2100 | 5 |  |
|  | Z2101 | 8 |  |
|  | Z2102 | 10 |  |
|  | Z2104 | 7 |  |
|  | Z2108 | 5 |  |
|  | Z2109 | 7 |  |
|  | Z2112 | 7 |  |
|  | Z2114 | 4 |  |
|  | Z2116 | 6 |  |
|  | Z2118 | 5 |  |
|  | Z2120 | 6 |  |
|  | Z2121 | 4 |  |
|  | Z2131 | 4 |  |
|  | Z2146 | 8 |  |
|  | Z2150 | 3 |  |
|  | Z2152 | 5 |  |
|  | **Total** | **476** |  |
|  | **Mean** | **5.23** |  |
|  | **Range** | **2-10** |  |
|  | **Median** | **5** |  |
